# Distinguishing between PTEN clinical phenotypes through mutation analysis

**DOI:** 10.1016/j.csbj.2021.05.028

**Published:** 2021-05-21

**Authors:** Stephanie Portelli, Lucy Barr, Alex G.C. de Sá, Douglas E.V. Pires, David B. Ascher

**Affiliations:** a Structural Biology and Bioinformatics, Department of Biochemistry, University of Melbourne, Melbourne, Victoria, Australia; b Systems and Computational Biology, Bio21 Institute, University of Melbourne, Melbourne, Victoria, Australia; c Computational Biology and Clinical Informatics, Baker Heart and Diabetes Institute, Melbourne, Victoria, Australia; d Baker Department of Cardiometabolic Health, Melbourne Medical School, University of Melbourne, Melbourne, Victoria, Australia; e School of Computing and Information Systems, University of Melbourne, Melbourne, Victoria, Australia; f Department of Biochemistry, University of Cambridge, 80 Tennis Ct Rd, Cambridge CB2 1GA, United States

**Keywords:** PTEN, PHTS, Genotype-phenotype correlations, Mutation analysis, Machine learning

## Abstract

Phosphate and tensin homolog on chromosome ten (PTEN) germline mutations are associated with an overarching condition known as PTEN hamartoma tumor syndrome. Clinical phenotypes associated with this syndrome range from macrocephaly and autism spectrum disorder to Cowden syndrome, which manifests as multiple noncancerous tumor-like growths (hamartomas), and an increased predisposition to certain cancers. It is unclear, however, the basis by which mutations might lead to these very diverse phenotypic outcomes. Here we show that, by considering the molecular consequences of mutations in PTEN on protein structure and function, we can accurately distinguish PTEN mutations exhibiting different phenotypes. Changes in phosphatase activity, protein stability, and intramolecular interactions appeared to be major drivers of clinical phenotype, with cancer-associated variants leading to the most drastic changes, while ASD and non-pathogenic variants associated with more mild and neutral changes, respectively. Importantly, we show via saturation mutagenesis that more than half of variants of unknown significance could be associated with disease phenotypes, while over half of Cowden syndrome mutations likely lead to cancer. These insights can assist in exploring potentially important clinical outcomes delineated by PTEN variation.

## Highlights

• This work links mechanistic effects of mutations and different phenotypes in PTEN.
• Distinct PTEN diseases lie on a spectrum of changes in protein stability and function.
• Computational analysis could accurately distinguish between PTEN phenotypes..

## Graphical abstract

**Figure:**
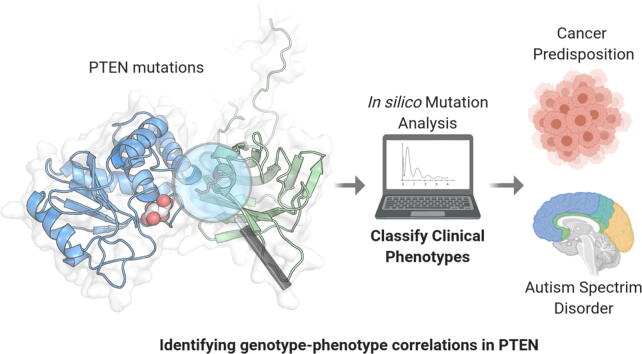

## 1 Introduction

Phosphatase and tensin homolog deleted on chromosome 10 (PTEN) is a dual-specificity phosphatase, and powerful tumor suppressor, with additional lipid dephosphorylation properties within the PI3K/AKT/mTOR signalling pathway. It is responsible for the dephosphorylation of PIP_3_ to PIP_2_, ultimately blocking cell division mediated by AKT. Independent of its PIP_3_ dephosphorylation activity, it is associated with the regulation of transcription, cell proliferation and genome maintenance [1]. PTEN activity is closely regulated by its subcellular localization, which is mediated by post-translational modifications (PTMs) including phosphorylation, SUMOylation and ubiquitination, as well as protein–protein interactions [1], [2].

**Fig. 1:**
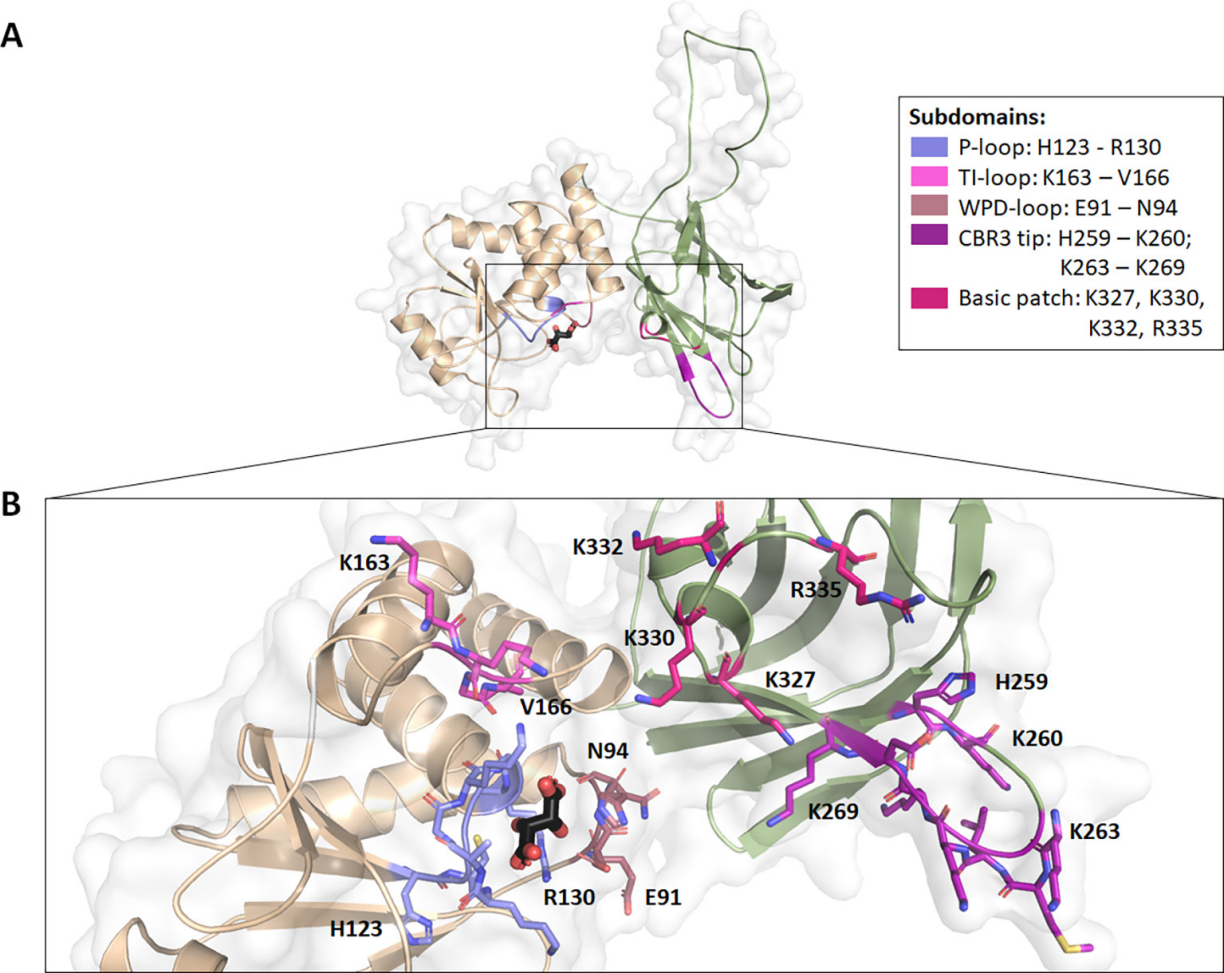
Main domains and subdomains present in PTEN. PTEN is primarily made up of two domains (A), the phosphatase domain (light orange) which comprises the P-, TI- and WPD- loops and the C2 domain (green) which comprises the membrane binding CBR3 tip and cɑ2 helix basic patch. The phosphatase is the site for PIP_3_ binding, shown in (B) bound to tartrate ion (black). (For interpretation of the references to colour in this figure legend, the reader is referred to the web version of this article.)

Structurally, PTEN is 403aa long and composed of two main domains (Fig. 1): (i) the phosphatase domain (N-terminus; residues 1–185), which contains the protein tyrosine phosphatase (PTP) conserved signature motif (HCXXGXXR) responsible for its dual-specificity phosphatase activity and lipid binding site [3], [4], and (ii) the C2 domain (C-terminus; residues 186–403), which contains a disordered loop spanning residues 286–309 [4], [5] (Fig. 1). The active site is essentially formed by the P-loop, which contains the HCXXGXXR motif, and the WPD- and TI-loop backbone atoms [4] (Fig. 1B). The TI loop is uniquely inserted in PTEN and is responsible for a large active site volume which permits PIP3 binding [4]. Parts of the WPD and TI loops are also present in the Phosphatase-C2 domain interface, which have been suggested to be important for overall folding, are highly conserved, and mutated in different cancers [4]. The C-terminal domain harbors the C2 domain, which contains the CBR3 loop responsible for PTEN attachment to the phospholipid membrane, with adequate phosphatase domain orientation to enable membrane-associated PIP_3_ binding [4]. This property is a result of the net + 5 charge, and two hydrophobic residues at the CBR3 tip, as well as a basic patch within the neighboring cɑ2 helix (Fig. 1B) [4]. Mutations in this domain were also associated with a reduction in PTEN’s tumor suppressor activity [4].

Missense mutations across the entire structure of PTEN have been associated with PTEN hamartoma tumor syndrome (PHTS) [6], an overarching condition with a broad range of phenotypes including different cancers and other tumorigenic states like Cowden syndrome [7], Bannayan-Riley-Ruvalcaba syndrome [8], Proteus [9] and Proteus-like [10] syndromes, to brain-related disorders such as macrocephaly, developmental delay and autism spectrum disorder (ASD) [11]. While tumor-related phenotypes have been attributed to changes in PIP_3_ dephosphorylation, the molecular consequences leading to ASD-related phenotypes remain unclear.

Initial efforts to understand PTEN-related disease mechanisms have focused on evaluating the effects of missense mutations on cellular fitness through an *in vivo* measurement of lipid phosphatase activity [12]. This revealed that the fitness effects of ClinVar pathogenic mutations and gnomAD population variants clustered in two distributions [12]. While this provided insight into the distinction between pathogenic and non-pathogenic mutations, differentiating between cancer-causing and ASD-causing phenotypes within the pathogenic class remains a challenge, primarily because of the limited numbers of reported ASD-causing mutations.

In an effort to address this, Smith and colleagues [13] looked at the effects of a limited subset of cancer-and ASD-causing mutations on PTEN’s conformational dynamics. They suggested that cancer-causing mutations (*n* = 6) exhibited higher connectivity to core PTEN nodes, and greater effects on interdomain interactions; while the ASD-causing mutations (*n* = 6) were focused at nodes near the CBR3 loop [11]. While this showed the potential for structural insights to delineate the different phenotypic outcomes of mutations in PTEN, it was based on a very limited subset of known PTEN disease mutations, and it was unclear how this might translate beyond those twelve mutations. Therefore, a more thorough analysis of the underlying molecular mechanisms across all clinically characterized variants is needed to provide a better understanding of overall disease etiology and how it can be treated.

We have previously shown that by considering the diversity of potential molecular consequences of a mutation on protein structure and function, it is possible to accurately predict mutations leading to cancer [14], [15], [16], [17], [18], different genetic diseases [19], [20], [21], [22], [23] and drug resistance [24], [25], [26], [27], [28], [29], [30], [31], [32], [33], [34], [35], [36]. Here, we therefore investigated the effects of mutations on protein stability, dynamics, activity, and molecular interactions across all clinically observed PTEN mutations till date, in order to identify molecular mechanisms driving the different clinical phenotypes in PTEN. Our analysis suggested that protein stability plays an important role in PTEN function and disease, where different pathologies displayed different residue-level interaction profiles and localized at different protein backbone environments. This suggests that PTEN stability and backbone conformation determines the subsequent interactions within biological pathways. A similar pattern was observed in lipid phosphatase activity, which is considered a proxy measure for cellular fitness, suggesting that protein stability and local residue interactions also mediate this functional effect.

## 2 Materials and methods

### 2.1 Dataset curation

Due to the wide spectrum of phenotypes manifested clinically resulting from missense PTEN mutations, data curation was carried out in sequential steps. Mutations conferring pathogenicity were initially extracted from ClinVar [37] (accessed July 2020), which classified them as ‘Pathogenic’ and ‘Likely Pathogenic’. To ascertain clinical involvement and increase confidence of assigned phenotypes, each mutation was cross checked with the literature, where only mutations identified directly from patients were kept for analysis. During this literature check, any mutations outside of the ClinVar dataset, which were similarly identified in clinical patients were also collected. Finally, to ensure that curation of pathogenic mutations was as comprehensive as possible, specific studies detailing large clinical PHTS, Cowden Syndrome (CS), or Bannayan-Riley-Ruvalcaba syndrome (BRRS) cohorts obtained from the Cleveland Clinic [38], [39], and a list of ASD and cancer mutations curated by Spinelli *et al.* [40], were used as a final check.

When present in the literature, clinical manifestations brought about by mutations were noted and used to assign a specific class (Suppl. Table 1). For the purposes of this study, the main pathogenic classes analyzed were ‘Cancer’ and ‘ASD’. Therefore, these phenotypes were prioritized even when co-occurring with other PHTS, CS and BRRS symptoms such as macrocephaly, gastro-intestinal polyps, café-au-lait marks, and thyroid dysfunction. While CS and its debated pediatric manifestation BRRS are linked to increased cancer risk, only mutations which were found in clinical cancer cases were assigned the ‘Cancer’ phenotype. Similarly, mutations in patients clinically presenting with ASD, developmental or speech delay or mental retardation were assigned the ‘ASD’ class. To further exhaust the search for PTEN mutations in ASD, mutations present in ASD-dedicated databases VariCarta [41] and SFARI [42] were similarly compared with the literature [11], [43], [44], [45], [46], [47], [48], [49], [50], [51], [52], [53], [54], [55], [56], [57], [58] and assigned the ‘ASD’ phenotype. Notably, during machine learning, a subset of ASD mutations which were only identified in ASD cases, without PHTS symptoms, were kept as a clinical validation test set.

Data curation revealed specific phenotypic manifestations within the PHTS condition. Specifically, patients having PHTS, CS or BRRS symptoms were sometimes observed to manifest neither cancer nor ASD (considered as ‘mild PHTS’), or both diseases (considered as ‘severe PHTS’). Ultimately, following consolidation of data from different sources, the interim ‘PHTS’ class was composed of ‘mild PHTS’ mutations not otherwise reported in a specific disease, and those which caused ‘severe PHTS’. A subset of ‘mild PHTS’ mutations identified in studies on CS and BRRS patient families were kept aside as a separate ‘CS’ class. Despite the lack of data accompanying these mutations with respect to cancer development, these mutations are considered ‘likely cancer-causing’ as it is known that CS is associated with increased cancer risk. The curation of data from different sources also identified overlaps across diseases. Mutations leading to both cancer and ASD in separate patients (identified from separate sources), were labelled ‘Both’.

Finally, following a thorough identification of pathogenic mutations within PTEN, any missense mutations present in the general population, as obtained from gnomAD [59] (accessed July 2020), which were not identified as pathogenic during data curation, were considered as ‘Non-Pathogenic’. This class also included one mutation in ClinVar which was classified as ‘likely Benign’. Further to that, ClinVar mutations which were classified as variants of unknown significance (‘VUS’) and were not identified in the clinical literature were kept aside as the ‘VUS’ class. A total of 229 missense mutations were grouped in specific pathogenic or non-pathogenic classes, while 294 mutations were retained in the VUS class.

**Fig. 2:**
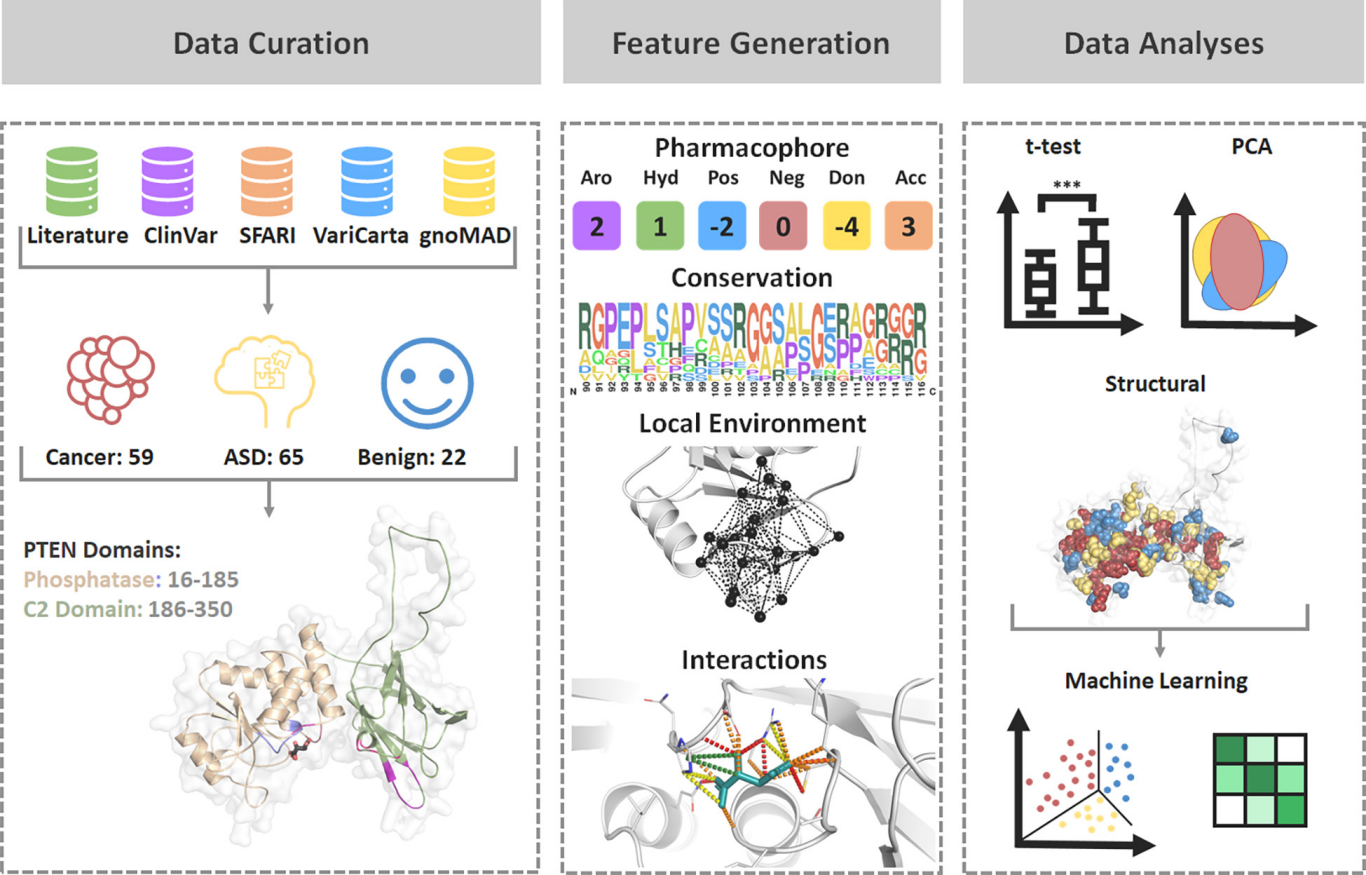
Methodology Pipeline followed in this study. Initial mutation curation was carried out to obtain Cancer (*n* = 59), ASD (*n* = 65) and Non-Pathogenic (*n* = 22; labelled as Benign in figure) mutations from five different sources. Data curation also involved processing of the experimental crystal structure to fill in missing residues and model the missing loop (286–309). *In silico* biophysical tools were then used to measure the effects of mutations on protein structure and function (Feature Generation), which was followed by different structural and statistical analyses and the development of a three-class prediction model.

For the purpose of this study, the main classes being compared were those describing ASD, Cancer, and Non-Pathogenic mutations. However, interim classes (PHTS, Both and CS) were also used in specific analyses, for possible insight into biological effects describing different mutation profiles, which can help delineate understandings of disease. A summary of the classes, description and use across different analyses is detailed in Table 1 and Fig. 2, while an account of all mutations curated in this work is detailed in Suppl. Table 1.

**Table 1:** Data curation and *in silico* analyses. The curation of data from different sources identified subsets of pathogenic mutations apart from ASD and Cancer, which were the main pathogenic classes of interest in this study. To best characterize the biological effects mediated by these mutations, all classes and subclasses served a purpose in our analyses, which consisted of qualitative structural analysis, statistical *t*-test, data visualization techniques and supervised machine learning (ML). The use of the subsets within each analysis is summarized.

Class	*n*	Description	Analyses
Cancer	59	Mutations present in cancer cases, irrespective of ‘mild PHTS’ and which have not been identified in ASD	Qualitative structural, statistical, data visualization, supervised ML
ASD	65	Mutations present in ASD patients, irrespective of ‘mild PHTS’ and which have not been identified in Cancer	Qualitative structural, statistical, data visualization, supervised ML: *n* = 43 for model development; *n =* 22 for clinical validation
PHTS	26	Mutations which either presented with overall PHTS symptoms, including CS and BRRS and no cancer/ASD (“mild PHTS”), or mutations manifesting in both diseases within the same patient (“severe PHTS”)	Qualitative structural, statistical, data visualization
Both	31	Mutations causing both Cancer and ASD, identified from separate patients	Qualitative structural, statistical, data visualization
CS	26	‘Mild PHTS’ mutations identified in CS/BRRS patients with no other phenotype identified	Qualitative structural, statistical, data visualization, supervised ML: identification of mutations increasing cancer risk
VUS	294	ClinVar classified ‘variants of unknown significance’ or ‘conflicting interpretations of pathogenicity’ which have not been identified in the pathogenic classes	Supervised ML: suggesting reclassification of VUS
Non-Pathogenic	22	Mutations present in the general population which have not been identified in the pathogenic datasets	Qualitative structural, statistical, data visualization, supervised ML

### 2.2 PTEN structural curation

The experimental crystal structures of PTEN bound to tartrate ion (TLA; PDB ID: 5BZZ [5]), and the vanadate ion (VO4; PDB ID: 5BZX [5]) were obtained from the RCSB Protein Data Bank. Both structures were of the full-length protein and had a good resolution (2.20–2.50 Å), unresolved N- and C- termini (1–13 and 352–403) and an unresolved flexible loop (residues 286–309). Prior to mutational analysis, the structures were preprocessed using Maestro (Schrodinger suites), and MODELLER [60] to fill in missing atoms and model the missing loop. The TLA-bound structure was used for all structural analyses, while the VO4-bound structure was only used to calculate changes in affinity to VO4 upon mutation, and associated distance between mutated residues and ion binding site.

### 2.3 Feature engineering

In order to quantify the different potential mechanistic effects of mutations to protein structure and function, a range of sequence- and structure-based properties were calculated using *in silico* biophysical tools (Fig. 2) in a manner previously described [26], [61]. A total of 101 features were calculated on the curated structures, which can be categorized into four classes describing (i) the local residue environment, (ii) non-covalent interactions, (iii) changes in active, binding and conserved sites, and (iv) predicted changes on protein stability and dynamics. A list of calculated features per category is summarized in Suppl. Table 2. For feature calculation requiring both wildtype and mutant structures (*e.g.*, differences in non-covalent interactions), homology modelling (using MODELLER [60]) was performed for every single-point mutation individually.

***Local residue environment.*** We calculated features describing the local residue environment, including backbone psi and phi angles, secondary structure (SST [62]), residue depth and relative solvent accessibility (using BioPython [63]), levels of disorder (using IUPRED [64]), protein fluctuation and deformation energies (using Bio3D [65]). As a measure of residue environment geometry and physicochemical properties, graph-based signatures were also calculated [66]. We have previously shown that graph-based signatures are a powerful approach to represent a protein 3D structure in order to predict the effects of mutations on protein stability [66], [67], [68], [69], [70] and interactions [66], [71], [72], [73], [74], [75], [76], [77].

***Interactions.*** Features describing PTEN interactions included changes in ligand affinity to TLA and VO4, and associated distances to ligand, which were calculated using mCSM-lig [76]. We also measured changes in local interactions upon mutation using Arpeggio [78] and described relevant molecular interactions as frequencies such as hydrogen bonds, pi-interactions and hydrophobic interactions. Changes in residue pharmacophore such as hydrogen bond donors and acceptors, were also calculated to reflect residue-level changes which can affect interactions.

***Functional changes.*** Since PTEN function is related to its conserved sites, we measured the rate of residue evolution through ConSurf [79], and analyzed the functional effect of each mutation using conservation-based features SIFT [80], SNAP2 [81] and PROVEAN [82] protein. Further to these, we measured the Missense Tolerance Ratio [83], [84], which accounts for rate of mutation under neutrality and evolutionary substitution matrices PAMs and BLOSUMs, which measure the statistical likelihood of a mutation to occur. Finally, an additional biological feature was obtained from Mighell *et al.* [12] which described the lipid phosphatase activity of each mutation, which is a function of cellular fitness.

***Changes in stability and dynamics.*** Changes in protein stability and dynamics upon mutation can play an important role in the emergence of different phenotypes [21], [23], [85], [86]. In this work we quantified these changes, also referred here as *in silico* biophysical measurements, using a range of well-established computational methods including mCSM-Stability [66], DUET [69], SDM [87], Dynamut [70] and ENCoM [88].

### 2.4 Qualitative structural and statistical analyses

The mutations within each phenotypic subset (ASD, Cancer and Non-pathogenic) were assigned to major molecular mechanisms of disease in a method similar to ones previously described [26], [89]. The *in silico* biophysical measurements of changes in ligand affinity, protein stability and protein dynamics were quantitatively compared for every mutation, and classified based on direction of change (*e.g.*, increased or decreased stability) and intensity (measured as the change in Gibbs Free Energy of folding or binding, ΔΔG, given in kcal/mol, and labelled as mild: 0.5 <= **|**ΔΔG**|** < 1; moderate: 1 <= **|**ΔΔG**|** < 2 or high: **|**ΔΔG**|** >=2) [89]. The overall mechanism assigned depended on the extent of mutational change across all mutational measurements. Proportions of overall mechanisms across the datasets were obtained to possibly shed light on the patterns underlying different phenotypic classes. Finally, a two-tailed Welch sample *t*-test [90] was carried out on all calculated features to identify stratifying features between all pathogenic (*n* = 207) and Non-Pathogenic (*n =* 22) mutations, and cancer-causing (*n =* 59) and autism-causing (*n =* 65) mutations using the t.test function in R (v.3.6.1) [91]. Similarly, to identify possible differences between interim classes, a two-tailed Welch sample *t*-test [90] was also carried out between the classes PHTS (*n* = 26) and ‘Both’ (*n* = 31), and PHTS (*n* = 26) and CS (*n* = 26). Features were considered significant if their associated *p*-value was < 0.05.

### 2.5 Data visualization techniques

A visual discernment between classes can highlight phenotype-distinguishing features. This was particularly important considering that a large number of features (n = 101), or dimensions, were generated to describe a small number of data points spread across the three main phenotype classes: ASD (n = 65), Cancer (n = 59) and Non-Pathogenic (n = 22). As the purpose of this analysis was to highlight potentially distinguishing features between these three main classes in lower dimensions, data visualization techniques were used on all features describing ASD, Cancer and Non-Pathogenic mutations. To visually compare the interim classes PHTS (n = 26), CS (n = 26) and ‘Both’ (n = 31) to the main phenotypes, these data points were plotted on the same 2D axes, and their clustering patterns observed.

Two different techniques were tested: Principal Component Analysis (PCA) [92] and uniform manifold approximation and projection (UMAP) [93], using R (v.3.6.1) [91] packages “cluster” and “umap”, respectively. These methods were chosen as they are based on different approaches: PCA is a linear approach, which focuses on maintaining data variance [92], while UMAP is non-linear, where the distances between individual data points are maintained in the visualization [93]. Testing out two fundamentally different approaches ensured that the data could be visually represented as comprehensibly as possible, while accounting for underlying correlations between data points. These techniques were carried out at different feature levels. When using all features, features contributing to the two visualized dimensions could help suggest protein properties underlying mechanisms of disease, particularly if the classes could be distinguished visually. This process was also carried out on the subset of features which presented high class stratification from the statistical analysis (n = 54, i.e., features that presented a significant distribution difference between classes), where a visual distinction between classes can again prioritize which features are most important. Based on this similar rationale, these techniques were also carried out on the final features identified following greedy feature selection. Visually inspecting how classes cluster at different feature levels could be considered as validation for the results from other methods, where an improvement in class distinction is expected upon lowering the number of features statistically, and through greedy feature selection.

### 2.6 Supervised learning

A predictive model was developed using supervised learning aiming to accurately distinguish between three classes of missense mutations arising in PTEN: ASD, Cancer and Non-pathogenic. This composes a multiclass classification problem, which can be tackled by different approaches. For simplicity, we opted to use the “transformation into binary” technique, assessing both OneVsOne (which performs a pairwise comparison of all classes) and OneVsRest (which accounts for the performance of one class compared to the remaining two) strategies during model development, both available within the scikit-learn (v.0.23.2) [94] “multiclass” package.

The predictive model was trained on the curated mutations describing clinical presence of ASD (n = 43) and Cancer (n = 59), and the Non-pathogenic (n = 22) mutations were derived from residual population variation. A subset (n = 22) of ASD mutations, which described mutations identified in ASD patients without other PHTS symptoms, was kept aside as a second clinical validation test, to verify the clinical utility of the final model. The remaining ASD data used (n = 43) described mutations causing ASD symptoms in conjunction with other PHTS symptoms, excluding cancer manifestation.

ASD (n = 43), Cancer (n = 59) and Non-pathogenic (n = 22) mutations were divided into a training set (70%; ASD: 32; Cancer: 39; Non-Pathogenic: 17) and a non-redundant blind test (30%; ASD: 11; Cancer: 20; Non-Pathogenic: 5), using the GroupShuffleSplit function within scikit-learn (v.0.23.2) [94], which retained the relative proportions of classes. Due to the smaller dataset curated for Non-Pathogenic mutations, training was also carried out at one level of oversampling for this class (n = 34), to establish a more balanced training set [95].

A range of classification algorithms available within the Python scikit-learn toolkit (v.0.23.2) [94] were assessed using default parameters: Gaussian Naïve Bayes, Support Vector Machines (kernel = ‘rbf’), K-nearest neighbor (k = 3), XGBoost (n_estimators = 300), Multilayer Perceptron, and the ensemble classifiers: Gradient Boosting (n_estimators = 300), ExtraTrees (n_estimators = 100), Random Forest (n_estimators = 300) and AdaBoost (n_estimators = 300). To minimize risk of overfitting, internal model validation was carried out during training through k-fold cross validation, at k = 3, 5 and 10. Briefly, this cross-validation splits the training dataset into k number of folds, and iteratively leaves one fold out as a test set. Due to the relatively small number of data points used for training, cross validation was carried out using the StratifiedKfold function within scikit-learn (v.0.23.2) [94], which ensured that each fold retained class proportions representative of the whole dataset, and that the final metrics were representative of the whole data. A bottom-up greedy feature selection approach was employed to minimize model complexity, as prevoiusly described [67], [68], [71], [72], [77]. Best performing models were selected based on the Matthew's Correlation Coefficient (MCC), which is a well-established and balanced metric not affected by class sizes. The best classifier was chosen out of 108 resultant models (Suppl. Table 6), based on consistent performance between cross-validation and blind test, and number of features. The final model was subjected to an additional clinical ASD dataset, which permitted the assessment of model applicability in the clinic.

## 3 Results

### 3.1 Curation of PTEN disease mutations reveals that cancer mutations cluster at the phosphatase domain

**Fig. 3:**
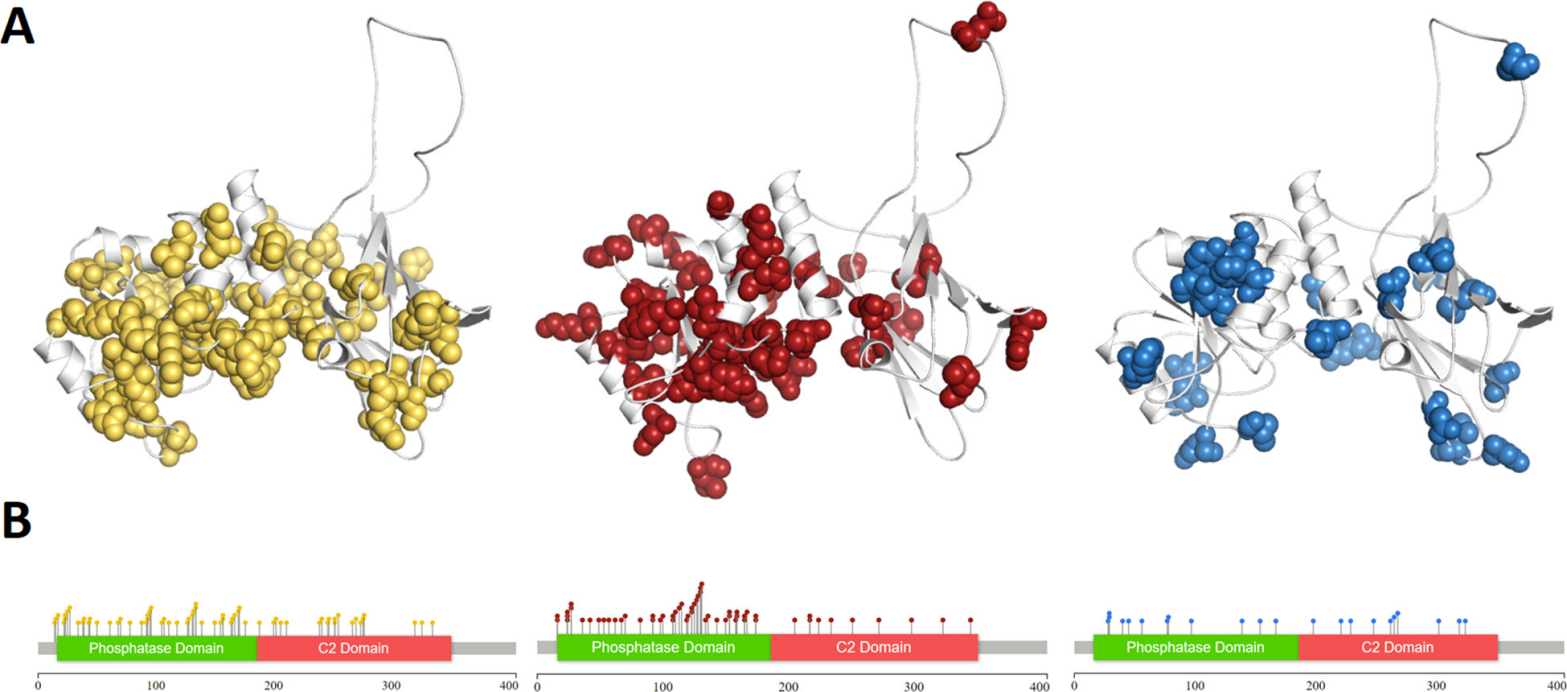
Mutation distribution. Differences in distribution of mutations across the three main phenotypes: ASD (yellow), Cancer (red) and non-pathogenic (blue) across protein structure (A), and gene (B). Cancer mutations are observed in higher concentrations within the phosphatase region, suggesting a direct effect on PIP_3_ dephosphorylation and subsequent tumor suppression. (For interpretation of the references to colour in this figure legend, the reader is referred to the web version of this article.)

The final curated dataset was obtained from ClinVar, gnomAD, SFARI and through the literature (in total 80 papers manually curated) and consisted of 65 ASD-causing, 59 cancer-causing and 22 Non-pathogenic mutations. In addition, two interim classes were defined, one labelled ‘PHTS’ (*n =* 26), which had the same phenotype in ClinVar with no additional details, and another labelled ‘Both’ (*n =* 31), which contained mutations associated with both ASD and cancer across the different sources. It is worth noting, however, that the major class of mutations obtained through ClinVar were variants of unknown significance (‘VUS’, *n* = 294), suggesting that disease etiology within PTEN is very complex and still poorly understood. Observing the spatial distribution of the main mutation classes within the gene and subsequent structure (Fig. 3) shows that, while mutations associated with either cancer or ASD were widely distributed across the whole gene, those causing cancer were more enriched within the phosphatase domain, which mediates its tumor suppressive function.

While it was previously proposed that ASD-causing variant effects were concentrated around the CBR3 tip, we observed that the spatial distribution of ASD-causing variants was not localized to a specific domain. This included ASD-causing variants in the PIP_3_-binding site residues: D92, H93 and Q171. Of these, D92 is required for protonation, while the other three residues are important for PIP_3_ binding [4]. When considering the phosphatase-C2 interdomain region, two ASD variants were observed in the C2 domain residue D252, which is involved in interdomain hydrogen-bond networks, and lies in a highly conserved region.

Cancer-causing mutations were predominantly found in the phosphatase domain, which is associated with the tumor-suppressing activity of PTEN, particularly in residues D92, H123, G127, K128, R130 and T167 which lie within the PIP_3_ binding site. Of these, K128 was reported to bind directly to PIP_3_, R130 is required for catalysis, H123 and G127 determine the conformation, and D92 is required for protonation. Some cancer mutations were also observed in the phosphatase interdomain region, in position Y174. This region is highly conserved within the protein, with neighboring residues S170 and R173 involved in interdomain hydrogen-bonding [4].

Finally, non-pathogenic mutations were observed at a lower frequency across the gene, with no specific domain localization. Only one variant was observed within the PIP_3_ binding site (at T167), and only two in the membrane binding region (at L265 and D268). Interestingly, no Non-pathogenic variants were observed in interdomain residues, suggesting that these residues play an important role in disrupting PTEN function and leading to both ASD and cancer.

### 3.2 Exploring the molecular consequences of PTEN mutations leading to disease

Analyzing the molecular and structural properties of mutations associated with disease (*n* = 207) to those identified as non-pathogenic (*n* = 22; Suppl. Table 3) revealed that most measures of conservation (as described in Section 2.3) showed a significant distinction between the two classes (ConSurf: *p =* 3.3e^−4^, PROVEAN: *p* = 2.0e^−5^, SNAP2: *p* = 1.6e^−6^, SIFT: *p* = 1.7e^−3^), suggesting that pathogenic mutations are more likely to be found at highly conserved regions, and would be associated with more deleterious fitness consequences. Consistent with this, pathogenic mutations were also more likely to be buried within the protein core (ResDepth: *p* = 1.1e^−4^, RSA: *p* = 2.2e^−3^), and to significantly destabilize the PTEN protein structure (SDM: *p* = 2.6e^−4^, DUET: *p* = 8.4e^−3^, mCSM-Stability: *p* = 0.03).

This disruption in protein stability may be explained through local environmental changes, where pathogenic mutations tended to localize at residues having a smaller backbone Psi angle (*p* = 0.04) and were enriched in mutations occurring from a wildtype Glycine (*p* = 4.8e^-5^), and to a mutant Proline (*p* = 2.7e^−5^). This suggests that a disruption in normal PTEN function in disease is mediated through changes in backbone conformation, consistent with a previous study by Smith *et al*. [13], suggesting different patterns of connectivity between cancer- and ASD-causing mutations. Other measures of local environment which highlight molecular differences underlying pathogenicity include graph-based signature features. Specifically, features describing the presence of polar residue atoms at varying distances from other polar (*e.g.* PP:11.00: *p* = 1.1e^−7^; PP:2.00: *p* = 9.8 e^−6^) or hydrophobic atoms (*e.g.* HP:11.00: *p* = 1.7e^−4^; HP:2.00: *p* = 1.6 e^−6^), were enriched for within the pathogenic mutation class. As polar residues mediate fundamental and specific interactions during molecular recognition, this distinct pattern observed for the pathogenic class suggests that these mutations are clustered at sites involved in specific molecular interactions.

Additionally, pathogenic mutations also exhibited differential interaction patterns at the residue level when compared to the non-pathogenic mutations. These residue-mediated interactions included wildtype residue polar interaction counts (*p =* 1.4e^−3^), which were higher at pathogenic mutation sites. These differences also explain the functional profiles observed through conservation-based features. In addition, while considering specific PTEN functions, we also observed that pathogenic mutations tended to be located close to the PIP_3_ binding site (distance to tartrate: p = 0.01) and leading to a significant reduction in ligand binding affinity (mCSM-lig, *p* = 0.02) and lipid phosphatase activity (*p* = 3.0e^−13^). This is consistent with a previous study [12] that suggested that lipid phosphatase activity was reduced by cancer and ASD mutations, and not by non-pathogenic ones.

### 3.3 Exploring the molecular differences of PTEN mutations leading to different pathogenicities

To better understand how pathogenic mutations in PTEN specifically lead to cancer (*n =* 59) or ASD (*n =* 65; Suppl. Table 3), we looked closer at the molecular and structural features describing these mutations. Interestingly, further to our observation on the role of backbone conformation in mediating pathogenicity, mutations causing cancer were observed to be significantly enriched (*p* = 0.05) in changes to Proline, while ASD mutations were significantly enriched in mutating from a Proline residue (*p* = 0.04). Further to this, at the residue interaction level, cancer mutations were observed to occur in residues mediating ionic interactions (*p* = 0.02) while ASD mutations clustered at ones mediating aliphatic amide-ring interactions (*p* = 6.0 e^−3^). These opposing properties highlight the involvement of mutant residue interactions in distinct molecular pathways and PTEN functions. These interactions and protein backbone profiles may further possibly explain the significantly different level of lipid phosphatase activity (*p* = 0.02) between classes, observed to be more disrupted via cancer mutations.

The trends underlying general PTEN pathogenicity, studied through the comparison of interim classes PHTS (*n =* 26) and ‘Both’ (*n* = 31; Suppl. Table 3) were less specific to those observed for the main pathogenic mutation classes, ASD and cancer. Interestingly, this comparison highlighted that mutations observed to lead to both diseases in separate patients, given by the ‘Both’ class, were present in more conserved regions (ConSurf: *p* = 0.05) and were enriched in aromatic substitutions (*p* = 0.04). On the other hand, mutations within the PHTS class, where the main phenotypes, if present, were occurring in the same patient, were observed to significantly occur from a Proline (*p* = 0.04), and lead to neutrally charged residues (*p* = 6.9 e^−3^). Similarly to what was observed when comparing pathogenic and non-pathogenic mutations, mutations in the ‘Both’ class were observed to occur closer to the PIP_3_ binding site (*p* = 4.1e^−3^), and were more enriched in neighboring polar residues given by graph-based signature features (*p =* 0.03), where mutations significantly increased hydrophobic interactions counts (*p* = 0.02) at the residue level. Collectively these results suggest that mutations found in both diseases separately (‘Both’) are more detrimental to fitness than those present in PHTS, given by a molecular profile closely related to pathogenicity effects. In comparing mutations causing CS with those present in the general condition PHTS, similar trends towards fitness costs were observed through the CS class, where these mutations reduced protein stability (DUET: *p* = 0.02; mCSM-Stability: *p* = 0.03; SDM: *p* = 0.02) and occurred at a greater backbone Phi angle (*p* = 3.5e^-3^) than PHTS mutations, again suggesting the role of backbone conformation in mediating disease.

When further considering the structural effects of mutations on protein stability (DUET), protein dynamics (Dynamut), ligand affinity and lipid phosphatase activity (Suppl. Table 4), we observed a general trend where non-pathogenic mutations were associated with neutral or mild effects, followed by mutations linked to ASD, which had a mix of mild and large molecular consequences, and finally Cancer mutations, which overall had the largest molecular effects. This suggests that PTEN mutations leading to cancer have higher fitness costs compared to those leading to ASD. The pattern was evident, for example, in changes in protein stability (Suppl. Tables 4 and 5), with 54.2% of Cancer, 52.3% of ASD and only 27.3% of non-pathogenic mutations predicted to decrease stability. By contrast, only 5.1% of cancer mutations increased protein stability as an overall effect, while 9.2% of ASD and 18.2% of non-pathogenic mutations were estimated to increase protein stability. When the same analysis was carried out on the interim classes ‘Both’, PHTS and CS, it was again observed that mutations present in the PHTS had milder fitness effects, particularly on protein stability, where 61.5% of mutations, compared to 71.0% in the ‘Both’ class, and 80.8% within the CS class were observed to reduce protein stability.

Collectively, these observations suggest that different pathogenic phenotypes within PTEN, even interim disorders, lie on a spectrum, where the main protein property involved seems to affect function through mutations in conserved regions, changes in core residues and lipid phosphatase activity. It is known that stability is also regulated by protein–protein interactions [1], suggesting that different stages of PTEN stability play a role in different biological pathways, hence leading to different diseases.

### 3.4 Using the structural consequences of PTEN mutations to distinguish distinct disease outcomes

To better understand the interplay of protein properties between the distinct disease states associated with PTEN mutations, we used data visualization techniques to analyze property distributions across both main (ASD, Cancer, Non-pathogenic) and interim classes (Both, PHTS and CS) and supervised machine learning approaches to assess our ability to predict the three main phenotypes observed.

**Fig. 4:**
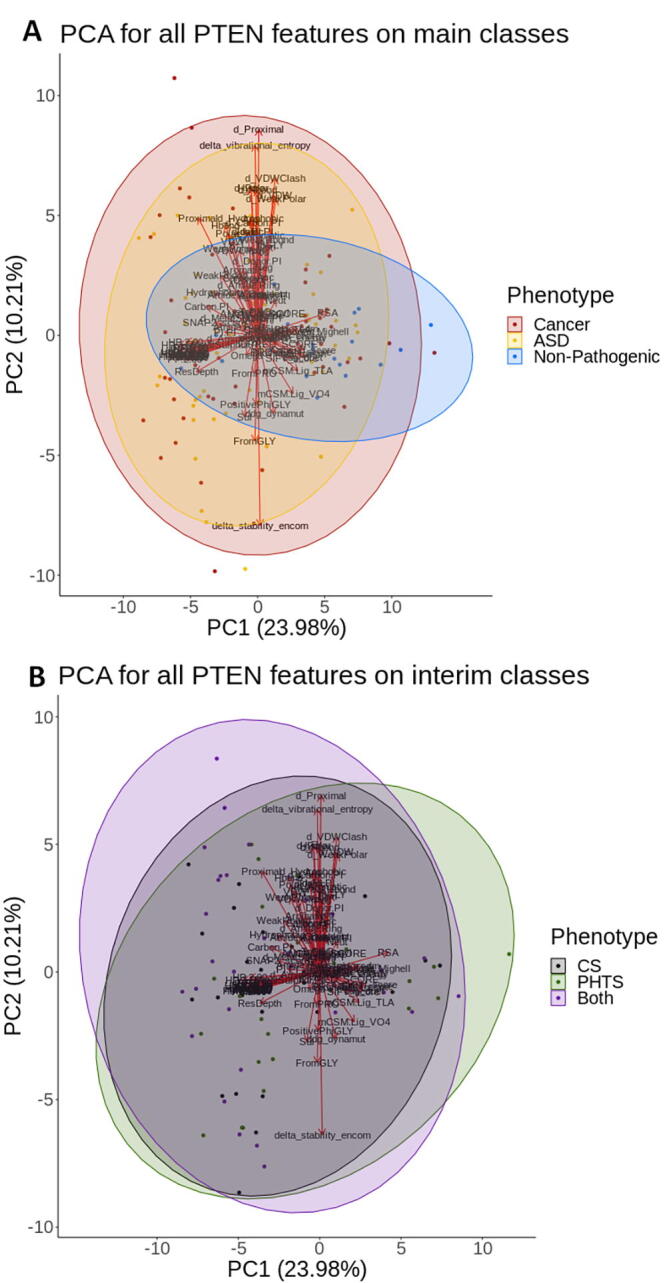
Principal component analysis plot on all features. When considering the main phenotypes (A), pathogenic classes ASD (yellow) and Cancer (red), were observed to overlap, while Non-pathogenic mutations (blue) mapped at distinct regions on the plot. A comparison of the interim classes (B) shows slight distinctions between Both (purple) and PHTS (green), while CS (grey) mutations lied in between. (For interpretation of the references to colour in this figure legend, the reader is referred to the web version of this article.)

Two different data visualization techniques: 2-component PCA and U-MAP, were evaluated across mutations labelled as ASD, Cancer and Non-Pathogenic and applied to the interim classes ‘Both’, PHTS and CS for observation. While U-MAP offered no visual insight into possible mutation distribution patterns across different protein properties (Suppl. Fig. 1), 2-component PCA consistently showed a distinction of the non-pathogenic class (Fig. 4, Suppl. Fig. 2; blue). The two principal components accounted for 34.2% of the variance observed within all the features. Despite this small variance, the main component PC1 (24.0%) was consistent with our previous analyses, and had significant contributions from mutation properties such as relative solvent accessibility (RSA), changes in protein stability (calculated by DUET, mCSM-Stability and SDM), lipid phosphatase activity, measures of conservation (ConSurf, PROVEAN, SIFT), changes in ligand affinity and distance to active site. These measures were all significant in distinguishing pathogenic from non-pathogenic mutations, which accounts for the visual distinction of the Non-pathogenic class from the rest. The second principal component (PC2; 10.21%) was composed of a measure of vibrational entropy change (ENCoM), which accounts for dynamic effects, but was primarily composed of residue level interactions such as changes in Polar, hydrogen bond and hydrophobic interaction counts. While interesting to note, these specific interaction types did not account for the distinct residue level interactions mediated by ASD and Cancer mutations observed from the statistical analysis, which can be visually observed in the plot through a direct overlap of these two classes. Interestingly, when plotting the interim classes on the same axes, common patterns between these classes, previously observed through other methods, have emerged. Specifically, mutations in the PHTS class (Fig. 4B; Suppl. Fig. 2; green), which were suggested by the structural analyses to have the mildest effects, mapped close to the Non-pathogenic mutations (Fig. 4A; blue). On the other hand, mutations in the ‘Both’ class (Fig. 4B; purple) shared a similar molecular property distribution to the Cancer mutations (Fig. 4A; Suppl. Fig. 2; red). This was in line with structural and statistical findings which suggested that mutations in the ‘Both’ class were the most disruptive. These results further highlight that different pathogenic classes within PTEN have spectral effects, where the resultant phenotype is an interplay between different pathway level effects.

Based on the consistent observations across different techniques, we employed supervised machine learning to assess the extent to which combinations of specific protein features can be used to distinguish between ASD, Cancer and Non-pathogenic mutations. Machine learning was carried out on all features generated, which included a graph-based signature representation of the 3-dimensional protein environment around the mutated residue [96]. The atoms, labelled as either hydrophobic or polar, were represented as nodes, with the edges capturing the molecular interactions between atoms. We have previously shown that graph-based signature representation of the mutation environment is a powerful and accurate approach to predicting the effects of mutations [61], [66].

During model development, different model parameters were tested in parallel across 108 runs (Suppl. Table 6) which included the presence or absence of oversampling for the non-pathogenic class, multiclass approaches *OneVsOne* and *OneVsRest*, different classification algorithms and different cross validation schemes (k = 3, 5, 10). When choosing the best performing model, greedy feature selection cut-offs prioritized consistent values between MCC cross-validation result and blind test. During this process, the number of features was kept to a minimum, in order to limit model complexity, with the AdaBoost algorithm being the best performing model under 10-fold cross validation, using oversampling of the least frequent class.

When comparing the two models obtained by the different multiclass approaches (*OneVsOne* and *OneVsRest*; Suppl. Table 7), it was observed that both models prioritized experimental lipid phosphatase activity, which describes PTEN function, a change in cation-Pi interaction counts and a graph-based signature feature describing the presence of two polar atoms within interacting distance (2 Å; Suppl. Table 7). Despite having more features (n = 11 compared to n = 7 in *OneVsRest*), the model based on the *OneVsOne* multiclass approach was chosen, as the MCC values obtained following cross-validation and subjection to a blind test were consistent and robust (MCC: 0.68). This model was additionally based on another graph-based signature detailing two polar residues within 6.5 Å of each other, relative solvent accessibility, MTR score, which is a measure of missense intolerance at a specific residue, and different residue level interaction counts involving Pi interactions (hydrogen bond donor-Pi, change in Pi-Pi interactions), aliphatic (amide-ring interactions) and aromatic residues. Further to that, this model accounted for mutations leading to Proline, which is known to change backbone conformation. Interestingly, most of these features were highlighted to be significant when distinguishing between pathogenic and non-pathogenic mutations (graph-based signatures, RSA), and between ASD and cancer-causing mutations (mutation to Proline, amide-ring interactions), while the functional lipid phosphatase activity was observed to contribute significantly to both stratifications from our *t*-test [90] analysis. These consistencies between analyses, further suggest model robustness, based on biologically discerning features among classes.

**Fig. 5:**
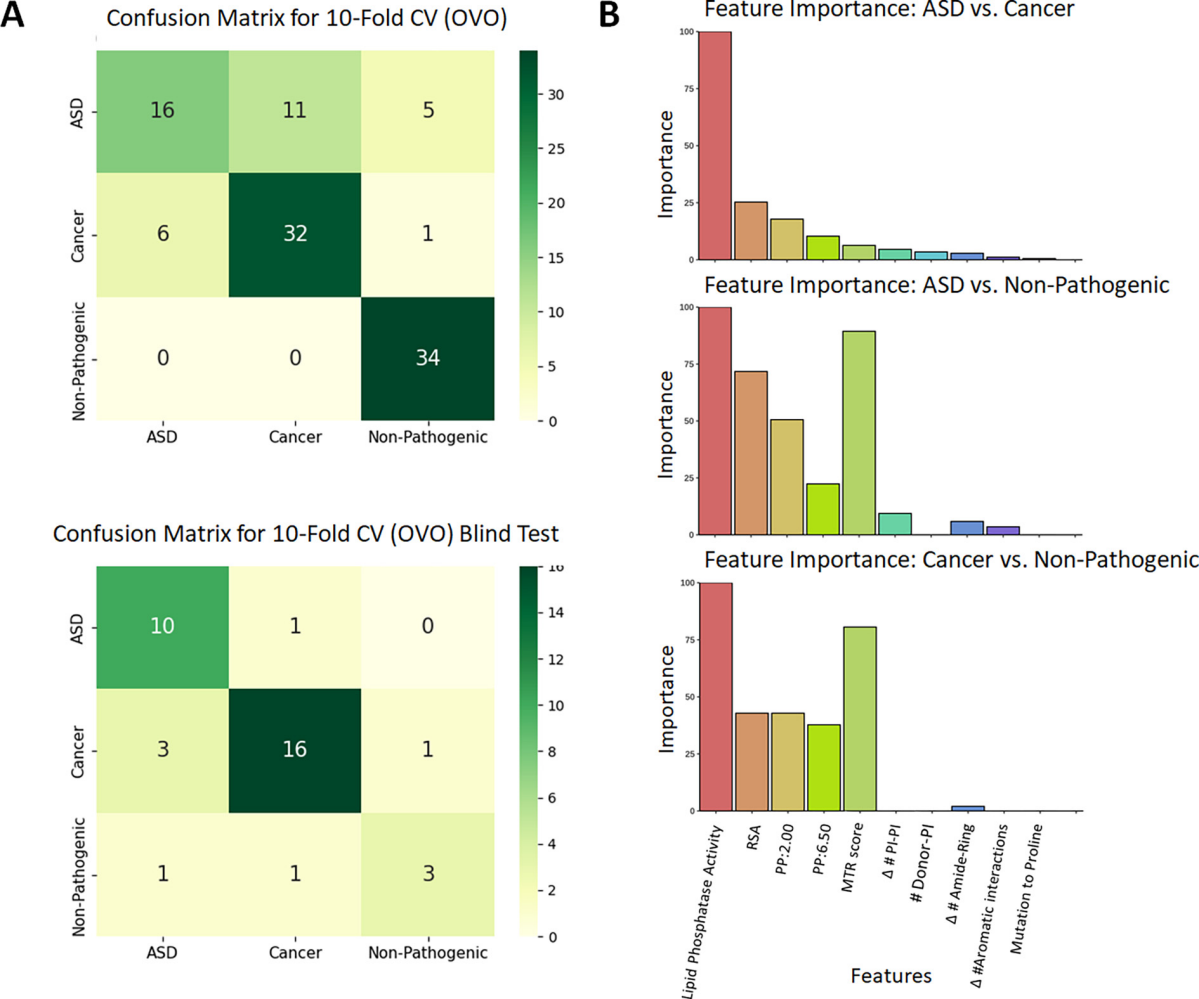
Metrics for the chosen model. Model was obtained after greedy feature selection, where the confusion matrices (A) calculated the three-class classifier using the *OneVsOne* method on the Adaboost algorithm, validated through 10-fold cross validation, with prediction performance of up to 0.68 MCC. Confusion matrices show correctly predicted data points per class, across the diagonal. (B) Observing the contribution of each feature within the estimators of our final model shows that MTR score and RSA are important in identification of disease variants, while changes in lipid phosphatase activity plays an important role in distinguishing between disease outcomes.

Further model assessment using other metrics, including those accounting for data imbalances: balanced accuracy and F1 score (with micro, macro and weighted averaging) confirmed model robustness through similar metrics obtained across the different validation methods. This is particularly important considering the small dataset used, and despite requiring oversampling for the non-pathogenic class. Finally, to quantify how well the model can detect each class, we calculated Recall per class via the confusion matrices (Fig. 5A, Table 2), which showed inconsistencies between cross validation and blind test, particularly for the ASD cohort. This suggests that the features, despite identified as significant through other methods within this study, may not fully encompass the complexities underlying ASD-mediating mutations. To further test the applicability of the model to predict ASD mutations, we checked the model performance on a held-out dataset (*n =* 22), which was not used in model development. This test also gave poor results, suggesting low confidence in clinical applicability. One possible reason beneath these ASD metrics could be the combination of PHTS phenotypes co-occurring with ASD in the dataset used for model development, particularly since the clinical validation test represented ASD-only mutations.

**Table 2:** Balanced metrics observed in final model. The final model performed similarly between cross validation and blind test, suggesting there is no inherent bias underlying predictions.

Validation method	MCC	B. acc	F1 (micro)	F1 (macro)	F1 (weighted)	Recall ASD	Recall cancer	Recall NP
10-fold CV	0.68	0.77	0.78	0.76	0.77	0.37	0.54	0.77
Blind test	0.68	0.77	0.81	0.77	0.81	0.91	0.41	0.60
ASD test	–	0.32	0.32	0.16	0.48	0.32	–	–

As our final model is based on OneVsOne binarization, three pairwise estimators contribute to the final phenotype prediction: ASD vs Cancer; ASD vs Non-Pathogenic and Cancer vs Non-Pathogenic. As a final analysis, we then observed the extent to which our final features (Suppl. Table 7) contribute to each pairwise problem. Fig. 5B shows that lipid phosphatase activity contributes highly to all pairwise estimators for classification. On the other hand, MTR score was a particularly important contributor to estimators involving the Non-Pathogenic class, reconfirming that pathogenic mutations are localized at functionally important regions of the gene. Interestingly, RSA and polar atom pairs within interacting distances were also involved in distinguishing either pathogenic class from the Non-pathogenic.

### 3.5 Exploring the potential disease landscape of PTEN using in silico saturation mutagenesis

In order to help guide analysis of novel variants, we performed *in silico* saturation mutagenesis using our best machine learning-trained model (Suppl. Tables 6 and 8; Suppl. Fig. 3). Looking at the distributions of the different predicted phenotypes across the gene, distinct patterns have emerged: predicted Cancer mutations predominantly occupied the phosphatase domain, non-pathogenic mutations were concentrated at the C2 domain, while ASD mutations distributed across the whole gene, concentrating at the interdomain region. This distribution is thought to reflect specific domain functions, particularly considering the predicted Cancer cluster at the phosphatase domain, which includes the PIP_3_ binding site. This also suggested that mutations within the dynamic loop of the C2 domain are most likely to be Non-pathogenic mutations. This suggests that our model might be able to detect specific mutations affecting membrane binding and consequent tumor suppressor activity.

Using the results from the saturation mutagenesis, we explored the predicted phenotypes of the Variants of Unknown Significance (VUS, *n =* 294) that had been curated. We observed that our model predicted 26.5% of these as ASD, 39.1% as Cancer and 34.4% as Non-Pathogenic. This suggests that nearly half of the previously uncharacterized variants could be associated with a disease phenotype, suggesting that further follow up work is needed to explore the potential clinical implications of these. Similarly, we also wanted to assess the cancer risk for a subset of mutations which were associated with CS/BRRS (*n =* 26), and found that our predictor considers 57.7% of these mutations to be cancer-causative. Clinically, this risk could be considered as part of patient management strategies.

## 4 Discussion

Missense mutations in PTEN lead to very diverse disease states, collectively referred to as PHTS. The main challenge in PTEN disease management is to differentiate and predict the effect of specific mutations within this gene, as treatment options and patient monitoring across cancer and ASD is very diverse. Further to this, we have observed that some mutations have been associated with both phenotypes across different clinical sources, highlighting that underlying mechanisms are affected by different traits and the interactions between them. Despite being germline mutations, manifestations may also differ between members of the same family, making the distinction between ASD and cancer phenotypes more complex, and suggesting a reason behind our lower predictive performance for ASD mutations.

Previous efforts to distinguish between disease phenotypes have looked at *in vitro* lipid phosphatase activity [12], a measure traditionally correlated with the cancer phenotype, and conformational dynamics approaches [13] on only a very small sample of cancer (*n =* 6) and ASD (*n* = 6) mutations. Despite the hypotheses presented, neither work offers an in-depth comparison of different mutational effects using multivariate protein properties, which can lead to a better, more holistic understanding of the biological problem. In this work, we have manually curated our mutations from different sources into three clinically confirmed phenotypes: ASD (*n =* 65), Cancer (*n =* 59) and Non-pathogenic (*n* = 22), making our dataset comprehensible enough to draw rational conclusions from our different analyses.

Through *in silico* biophysical measurements describing different protein properties, we have sought to identify the molecular basis behind why specific mutations lead to one phenotype and not the other. Using different data analysis techniques, the effect of mutations on protein stability, as well as their localization in buried and conserved regions have consistently been observed to lead to pathogenicity. In comparing ASD-causing and cancer-causing mutations, we observed that different interaction profiles at the residue level correlated with protein backbone conformation effects, highlighting that protein conformation may be responsible for different diseases. A similar pattern was observed when considering *in vitro* lipid phosphatase activity, which could differentiate across all classes: ranging from the most detrimental (cancer) to hypomorphic (ASD), to wild type (non-pathogenic) function. We also analyzed mutations leading to both ASD and Cancer in different patients (‘Both’ class), it was observed that these mutations were a more disruptive ‘interim’ class, suggesting a molecular basis for age-related mutational penetrance between those who develop ASD and those who develop cancer. On the other hand, mutations in patients with co-occurring PHTS symptoms, including both ASD and cancer within the same patient, were observed to have less disruptive effects on protein properties in this study. These results collectively show that pathogenicity within PTEN may occur as a spectrum, consistent with the hypothesis proposed by Mighell *et al.* [12], with the most intense pathogenic mutations leading to cancer, and ASD causing mutations lying between the two extremes (cancer and non-pathogenic).

Using these structural insights, we have developed a three-class prediction model, trained through supervised machine learning. During development, our model was better able to detect cancer-causing and non-pathogenic mutations, where a reduced applicability to ASD may be due to co-occurrent (mild) PHTS conditions. Subjecting the model to two sets of validation: 10-fold cross validation and validation through a blind test showed comparable results across different balanced metrics, implying that the model has not been overfit on the data it has been trained on. Our final model is primarily composed of local, functional and interaction-describing features, suggesting that differences in phenotype manifestation lie predominantly at the molecular level. One possible avenue for further improvement in our final model, however, is the inclusion of epistatic effects through protein–protein interaction features, as these are known to be regulatory mechanisms driving PTEN action, and further optimizing the model towards detection of ASD-only mutations in younger patients.

Applying this model to an *in silico* saturation mutagenesis approach described a phenotypic landscape which linked back to the specific domain functions, primarily in the localization of the predicted cancer-causing mutations. In observing the predictions on the VUS data points we saw that there is potential for reclassification of up to 65.6% of these mutations into cancer or ASD, warranting more specific patient monitoring for those patients. Further to that, we also tested for cancer-risk in a subset of mutations from CS/BRRS patients, where 58% of mutations were predicted to lead to cancer. Despite the small dataset used for training, the metrics describing our final model strongly suggest a potential for clinical utility, particularly at guiding protocols for patients with VUS and CS. Finally, while PHTS is also commonly mediated through truncating mutations, a distinction of the different phenotypes brought about by missense mutations could inform therapeutic development for the underlying pathologies.

## 5 Conclusions

Mutations in PTEN are associated with a range of complex disease phenotypes, which have proven hard to untangle. By considering the structural and functional consequences of mutations in PTEN, we have shown that disease phenotypes can be accurately predicted. This also revealed key underlying molecular drivers of disease outcomes, with decreases in protein stability and phosphatase activity associated with disease. Interestingly, the severity of these effects appeared to correlate with phenotype, with the most drastic effects linked to cancer, mild reductions linked to ASD and non-pathogenic mutations showing minimal changes. Using these insights, we have identified that more than half of currently assigned VUS could be disease associated, and used our model to predict the phenotypic outcomes of all possible mutations. This will be a valuable resource to further explore the role of mutations in PTEN and their links to patient outcomes and treatments.

## CRediT authorship contribution statement

S.P. was responsible for data curation, structural and statistical analysis, machine learning and manuscript preparation. L.B. assisted with data curation. A.G.C.d.S. assisted with machine learning. D.E.V.P. assisted with supervision of machine learning. D.B.A. conceived, designed and supervised all aspects of the project. All authors assisted with manuscript writing.

## Declaration of Competing Interest

The authors declare that they have no known competing financial interests or personal relationships that could have appeared to influence the work reported in this paper.

## Footnotes

Appendix A Supplementary data to this article can be found online at https://doi.org/10.1016/j.csbj.2021.05.028.
