# Supplementary material for: Distinguishing between PTEN clinical phenotypes through mutation analysis
Source: Comput Struct Biotechnol J. 2021 May 21;19:3097–109. doi: 10.1016/j.csbj.2021.05.028 (PMC8180946; doi:10.1016/j.csbj.2021.05.028)
Supplement: Supplementary data 1 [file mmc1.docx]

**Distinguishing between PTEN clinical phenotypes through mutation analysis**

Stephanie Portelli^1,2,3^, Lucy Barr^1,2,3^, Alex G.C. de Sá^1,2,3,4^, Douglas E.V. Pires^1,2,3,5*^, David B. Ascher^1,2,3,6*^

^1^ Structural Biology and Bioinformatics, Department of Biochemistry, University of Melbourne, Melbourne, Victoria, Australia
^2^ Systems and Computational Biology, Bio21 Institute, University of Melbourne, Melbourne, Victoria, Australia
^3^ Computational Biology and Clinical Informatics, Baker Heart and Diabetes Institute, Melbourne, Victoria, Australia
^4^ Baker Department of Cardiometabolic Health, Melbourne Medical School, University of Melbourne, Melbourne, Victoria, Australia

^5^School of Computing and Information Systems, University of Melbourne, Melbourne, Victoria, Australia
^6^ Department of Biochemistry, University of Cambridge, 80 Tennis Ct Rd, Cambridge CB2 1GA

*To whom correspondence should be addressed D.B.A. Tel: +61 90354794; Email: [david.ascher@unimelb.edu.au](mailto:david.ascher@unimelb.edu.au). Correspondence may also be addressed to D.E.V.P. [douglas.pires@unimelb.edu.au](mailto:douglas.pires@unimelb.edu.au).

**Supplementary Information**


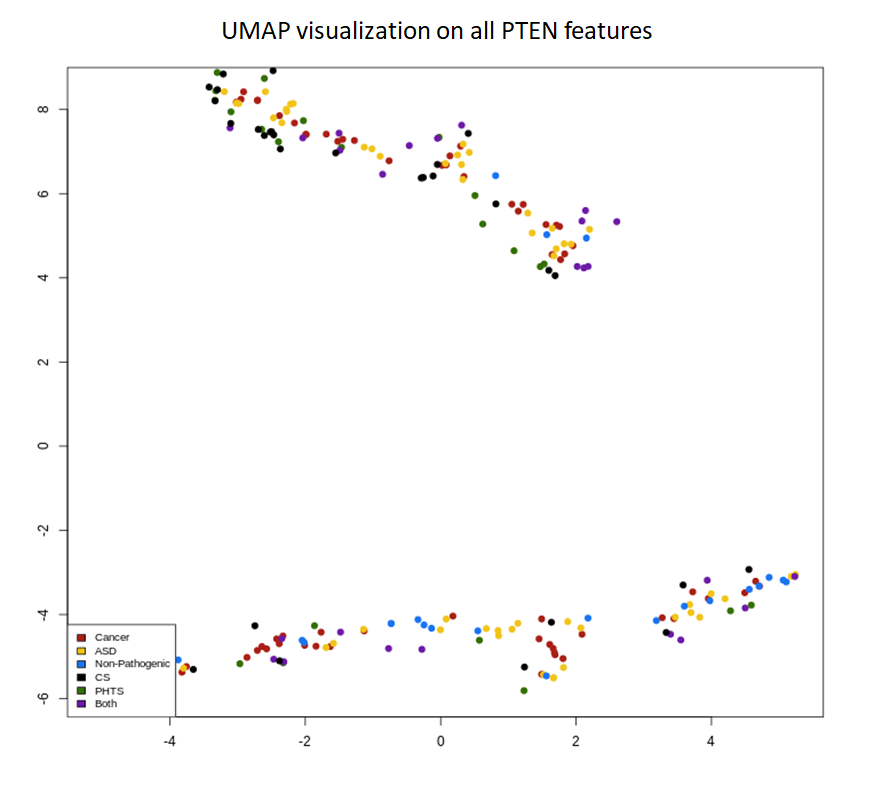


**Suppl. Fig. 1: Data visualization UMAP plot.** Unlike PCA, using UMAP to visually interpret the different classes did not prove useful, as different data clusters were heterogenous in phenotype.


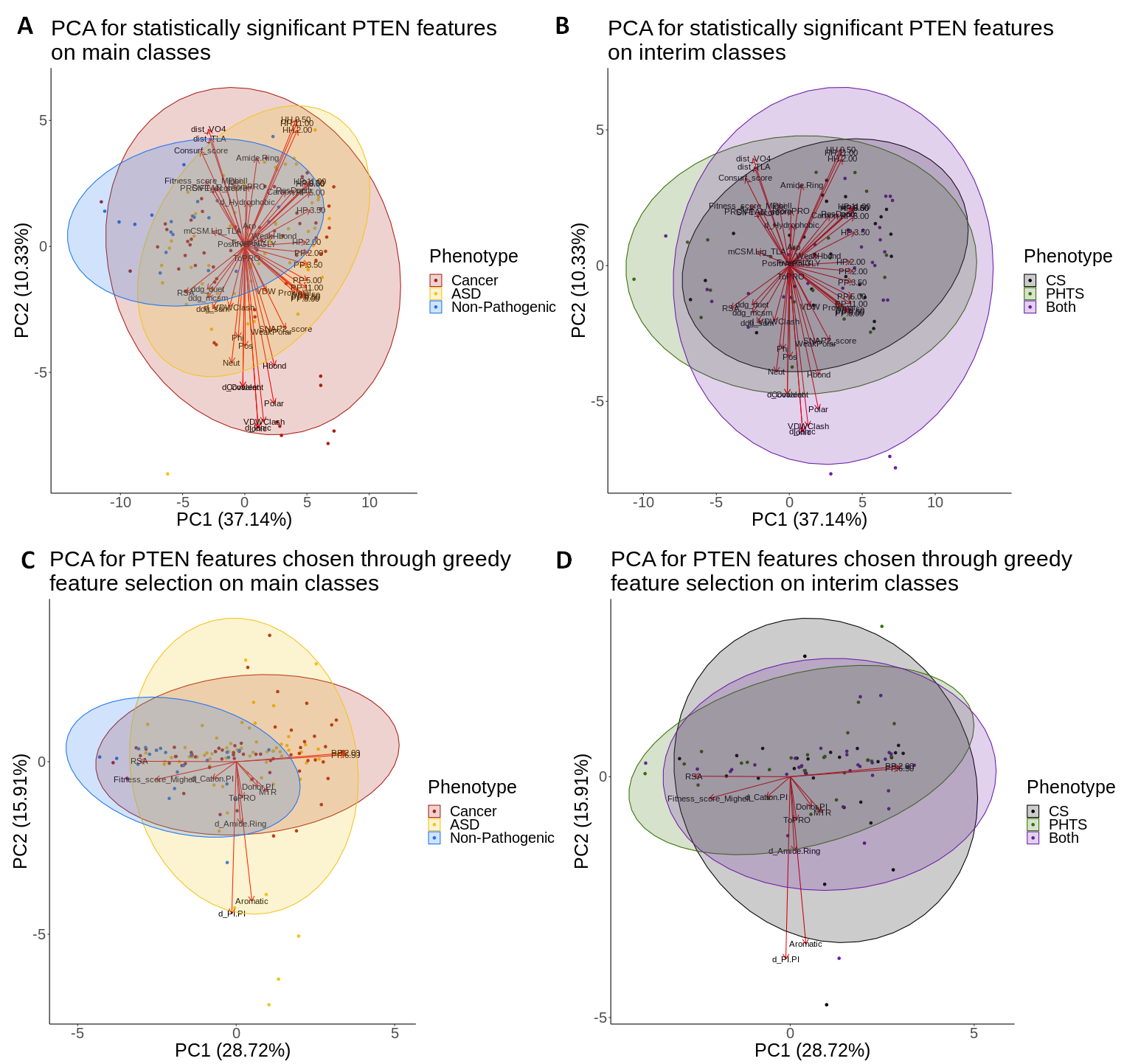


**Suppl. Fig. 2: Data visualization PCA plots.** PCA plots using significant features (*p-*value < 0.05) on the main classes (A) and the interim classes (B) shows an improved distinction between classes, where the more disruptive phenotype of the Both class (purple) can be observed to cluster similarly to the cancer class (red), while the milder PHTS (green) class distributes similarly to the non-pathogenic class (blue). This similar pattern is again observed when visualizing data on the final features used within our predictive tool, where a better distinction between the three main classes (C) is observed, and comparative interim class patterns (D) are retained.


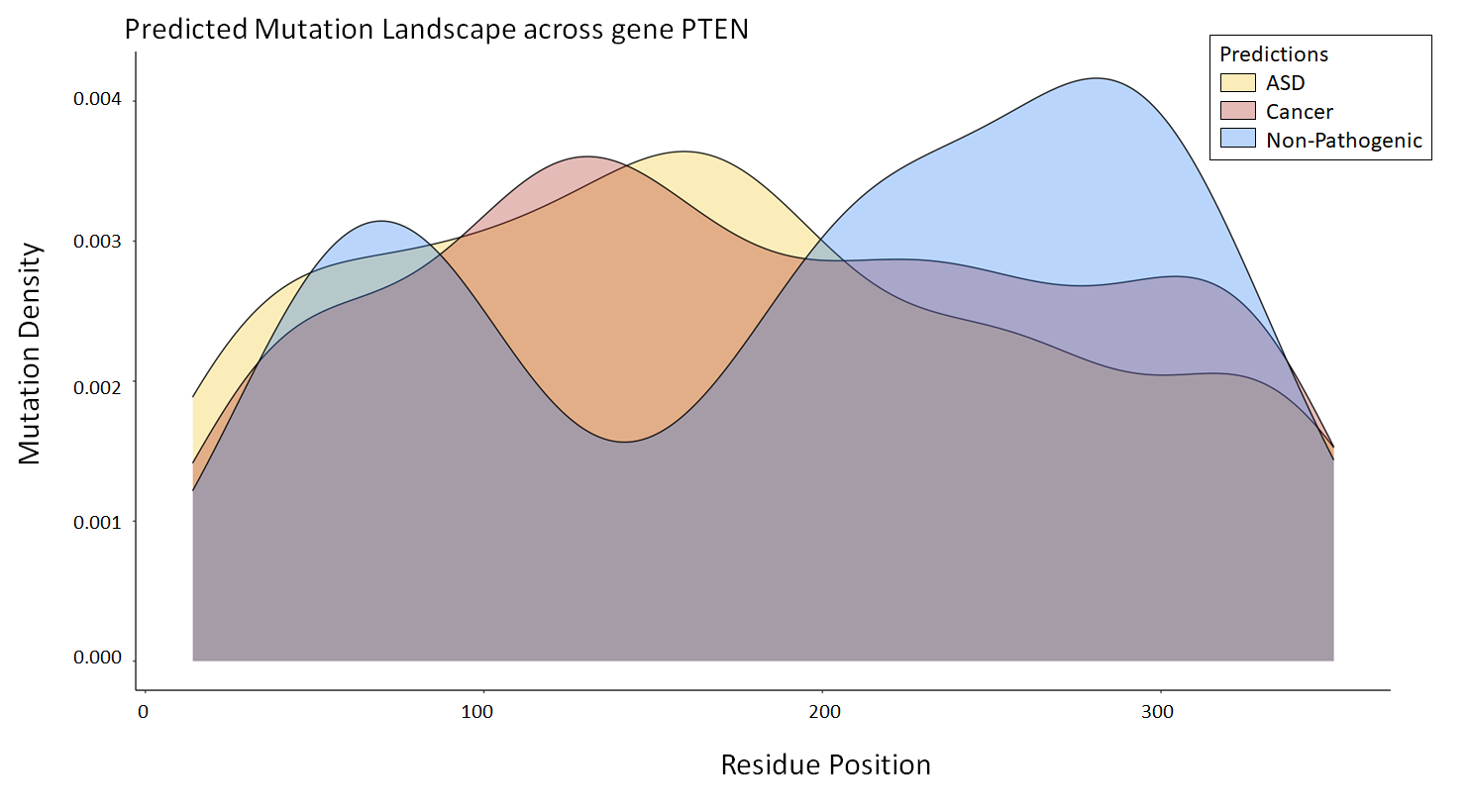


**Suppl. Fig. 3: Predicted mutational landscape across the PTEN gene.** Predicted mutation densities from an *in silico* saturation mutagenesis (Suppl. Table 8) suggest that cancer mutations primarily cluster at the phosphatase domain, while Non-pathogenic mutations peak at the flexible loop within the C2 domain.

**Suppl. Table 1: List of curated PTEN mutations with respective phenotypes and molecular effects.** Literature and other mutational sources used during data curation are listed, along with phenotypic comments seen in the literarture. Within the phenotype column, ASD2 mutations are those kept aside for validation during machine learning; CS refers to Cowden Syndrome or BRRS; PHTS1 refers to 'mild PHTS' and PHTS2 refers to 'severe PHTS'; while other phenotypes are as described in text.

| Mutation | PHTS/Cancer | ASD | Clinvar class | gnoMAD | Reference | Comments | Phenotype |
| --- | --- | --- | --- | --- | --- | --- | --- |
| R14G | PHTS1 | Yes | Pathogenic | No | Frazier et al. 2014^1^;  Hansen-Kiss et al. 2017^2^; Mighell et al. 2020^3^ | HK:Patients tested for: PHTS; CS; BRRS; pateint showed ASD; DD; dermatological conditions; Mighell: CC cohort patient showed ASD/DD; other patient showed dermatological conditions | ASD |
| R15I | - | Yes | - | No | Lionel et al. 2017^4^ | Present in SFARI | ASD2 |
| R15S | Cancer | Yes | Pathogenic/Likely pathogenic | No | Pilarski et al. 2001^5^;  Vanderver et al. 2014^6^ | Pilarski: Study on CS and BRRS phenotypic patients; Vanderver: MRI screen on patients with PHTS; patient observed to have DD; Macro | Both |
| Y16D | Cancer | No | Likely pathogenic | No | Mighell et al. 2020^3^ | identified in CC cohort; male patient with breast carcinoma and different adenomas | Cancer |
| Y16H | Cancer | No | Likely pathogenic | No | Ngeow et al. 2014^7^;  Mighell et al. 2020^3^ | Ngeow: Patients recruited for CS or CS-like phenotypes; present in secondary malignant neoplasms; Mighell: identified in CC cohort; presented with breast; endometrial and different cancers | Cancer |
| Q17E | PHTS1 | Yes | Uncertain significance | No | Mighell et al. 2020^3^ | Found in CC cohort with ASD | ASD |
| D22E | - | Yes | - | No | Buxbaum et al. 2007^8^ | Present in SFARI | ASD2 |
| D22G | Cancer | No | Uncertain significance | No | Pilarski et al. 2011^5^ | Study on CS and BRRS phenotypic patients | CS |
| L23F | PHTS1 | Yes | - | No | Mighell et al. 2020^3^ | Found in CC cohort; patient presented with DD; Macro and global overgrowth | ASD |
| L23V | PHTS1 | Yes | - | No | Mighell et al. 2020^3^ | Found in CC cohort; patient had ASD; DD; Macrocephaly | ASD |
| D24G | Cancer | No | Pathogenic/Likely pathogenic | No | Tan et al. 2011^9^;  Ngeow et al. 2014^7^;  Mighell et al. 2020^3^ | Tan: identified in cohort with relaxed CS criteria; Ngeow: CS/CS-like syndromes - present in secondary malignant neoplasms; Mighell: identified in CC cohort; patient presented with multiple cancers including breast and endometrial | Cancer |
| D24H | Cancer | No | Pathogenic | No | Tan et al. 2011^9^;  Ngeow et al. 2014^7^;  Mighell et al. 2020^3^ | Tan: identified in cohort with relaxed CS criteria; Ngeow: CS/CS-like syndromes - present in secondary malignant neoplasms; Mighell: identified in CC cohort; patient presented with multiple cancers including breast and endometrial | Cancer |
| D24N | Cancer | No | Likely pathogenic | No | Mighell et al. 2020^3^ | identified in CC cohort; male patient with breast carcinoma and different adenomas | Cancer |
| D24Y | Cancer | Yes | Pathogenic | No | Celebi et al. 1999^10^;  Bubein et al. 2013^11^ | Celebi: In patients with BRRS: Patient exhibited Macrocephaly; DD; p.f. breast lipoma; Bubien: identified in CS patient with basal cell cancer | Both |
| L25F | PHTS1 | Yes | Uncertain significance | No | Mester et al. 2012^12^;  Ciaccio et al. 2018^13^;  Tan et al. 2011^9^;  Mighell et al. 2020^3^ | Tan: Patients met relaxed criteria for CS; Mighell: patient had ASD and different BRRS symptoms, also present in SFARI | ASD |
| T26P | Cancer | Yes | Likely pathogenic | No | Tan et al. 2011^9^;  Pilarski et al. 2011^5^;  Mighell et al. 2020^3^ | Tan: identified in cohort with relaxed CS criteria; Pilarski: Study on CS and BRRS phenotypic patients; Mighell: variable delay; Lhermitte-Duclos disease | Both |
| Y27C | PHTS1 | Yes | Likely pathogenic | No | Vanderver et al. 2014^6^ | MRI screens in patients with PHTS; patient observed to have DD; Macro | ASD |
| Y27N | Cancer | No | - | No | Ngeow et al. 2014^7^;  Mighell et al. 2020^3^ | Ngeow: Patients with CS or CS-like syndromes having risk of secondary malignant neoplasms; Mighell: patient presented with endometrial and ovarian cancer | Cancer |
| Y27S | Cancer | No | Pathogenic | No | Davies et al. 1999^14^ | identified in clinical gliomas; acquired somatic mutation | Cancer |
| I28T | - | No | - | Yes | - | - | Non-pathogenic |
| Y29F | - | No | - | Yes | - | - | Non-pathogenic |
| Y29N | - | No | - | Yes | - | - | Non-pathogenic |
| P30L | PHTS1 | No | - | No | Bubien et al. 2013^11^ | Found in CS patient with no cancer | PHTS |
| I32N | Cancer | No | - | No | Tan et al. 2011^9^;  Pilarski et al. 2011^5^ | Patients met relaxed criteria for CS; Study on CS and BRRS phenotypic patients | CS |
| A34D | Cancer | No | - | No | Tan et al. 2011^9^;  Pilarski et al. 2011^5^;  Marsh et al. 1999^15^ | Patients met relaxed criteria for CS; Study on CS and BRRS phenotypic patients; study on CS and BRRS patients | CS |
| A34V | - | Yes | Uncertain significance | No | Monies et al. 2019^16^ | Present in SFARI | ASD2 |
| M35T | Cancer | No | Likely pathogenic | No | Tan et al. 2011^9^ | identified in cohort with relaxed CS criteria | CS |
| M35V | Cancer | Yes | Likely pathogenic | No | Tan et al. 2011^9^;  Hagelstrom et al. 2016^17^; Mighell et al. 2020^3^ | Tan:identified in cohort with relaxed CS criteria; Hagelstrom: identified in CS male patient with lymphoma and breast cancer; Mighell: different patients in CC cohort presented with PHTS symptoms and ASD | Both |
| G36E | Cancer | No | Pathogenic | No | Bilbao et al. 2006^18^ | identified in endometrial cancer patients | Cancer |
| G36R | Cancer | Yes | Pathogenic | No | Pilarski et al. 2011^5^;  Celebi et al. 2000^10^ | Pilarski: Study on CS and BRRS phenotypic patients; Celebi: identified in CS patient; Nickel: identified in glioblastoma patient; Mighell: patient presented with CS/BRRS symptoms but no cancer | Both |
| P38H | - | Yes | - | No | Klein et al. 2013^19^ | - | ASD2 |
| A39P | Cancer | No | - | No | Tate et al. 2008^20^ | Found in CS patient | CS |
| A39T | PHTS1 | Yes | - | No | Mighell et al. 2020^3^ | Found in CC cohort; patient presented with DD; Macro and ASD | ASD |
| E40D | - | No | - | Yes | - | - | Non-pathogenic |
| L42P | Cancer | No | Uncertain significance | No | Davies et al. 1999^14^ | Found in glioma tumors; acquired somatic mutation | Cancer |
| E43K | PHTS1 | Yes | Uncertain significance | No | Tan et al. 2011^9^;  Mighell et al. 2020^3^ | Tan:Patients met relaxed criteria for CS; Mighell: CC cohort; patient exhibited DD; dysmorphia; macrocephaly | ASD |
| G44D | PHTS1 | Yes | Pathogenic | No | Varga et al. 2009^21^;  Hansen-Kiss et al. 2017^2^; Vanderver et al. 2014^6^ | HK: Patients tested for: PHTS; CS; BRRS; Pateint showed ASD; dermatological conditions; Vanderver: MRI scans on patients with PHTS; patient had DD; Macro | ASD |
| V45I | - | No | - | Yes | - | - | Non-pathogenic |
| R47G | PHTS1 | No | Pathogenic/Likely pathogenic | No | Marsh et al. 2001^22^ | identified in patient with CS; patient showed GI polyps; fibroadenomas of the breast; Hashimoto's thyroiditis; papules but no cancer | PHTS |
| N48K | Cancer | No | Likely pathogenic | No | Tan et al. 2011^9^;  Pilarski et al. 2011^5^;  Mighell et al. 2020^3^ | Tan: identified in cohort with relaxed CS criteria; Pilarski: Study on CS and BRRS phenotypic patients; Mighell: CC cohort patient exhibited CS/BRRS symptoms | CS |
| N49S | Cancer | No | Uncertain significance | No | Mighell et al. 2020^3^ | Found in CC cohort; patient had different ductal cancers | Cancer |
| I50T | PHTS1 | Yes | Uncertain significance | No | Saskin et al. 2017^23^;  Vanderver et al. 2014^6^ | Vanderver: MRI screens in patients with PHTS; patient observed to have developmental delay and macrocephaly; also present in SFARI | ASD |
| D52G | Cancer | No | - | No | Tan et al. 2011^9^ | Patients met relaxed criteria for CS | CS |
| V53I | Cancer | No | - | No | Mighell et al. 2020^3^ | Found in CC cohort; patient had ductal breast cancer | Cancer |
| F56C | - | No | - | Yes | - | - | Non-pathogenic |
| L57W | Cancer | No | Likely pathogenic | No | Myers et al. 1997^24^ | identified in glioblastoma | Cancer |
| H61D | PHTS1 | No | - | No | Reardon et al. 2001^25^ | Vater association - different phenotype | PHTS |
| H61R | Cancer | Yes | Pathogenic/Likely pathogenic | No | Hansen-Kiss et al. 2017^2^;  Tan et al. 2011^9^;  Pilarski et al. 2011^5^;  Ngeow et al. 2014^7^;  Mighell et al. 2020^3^ | HK: Patients tested for: PHTS; CS; BRRS; Pateint showed ID; dermatological conditions; BRRS; Tan: identified in cohort with relaxed CS criteria; Pilarski: Study on CS and BRRS phenotypic patients; Ngeow: CS/CS-like syndromes - present in secondary malignant neoplasms; Mighell: ductal breast and intraductal carcinoma | Both |
| H61T | Cancer | No | - | No | Tan et al. 2011^9^ | Patients met relaxed criteria for CS | CS |
| H61Y | PHTS1 | Yes | - | No | Monies et al. 2019^16^;  Mighell et al. 2020^3^ | Mighell: Found in CC cohort in PHTS patient with variable delay; also present in SFARI | ASD |
| K62R | Cancer | No | - | No | Nassif et al. 2014^26^ | Found in colorectal cancer | Cancer |
| Y65C | Cancer | Yes | Uncertain significance | No | Vanderver et al. 2014^6^;  Nassif et al. 2014^26^ | Vanderver: MRI on PHTS patient shows DD/ASD; Nassif: identified in colorectal cancer | Both |
| I67R | Cancer | No | - | No | Marsh et al. 1998^27^;  Bubien et al. 2013^11^ | Marsh: Found in CS family; Bubien: Found in CS + endometrial cancer | Cancer |
| Y68C | Cancer | Yes | Pathogenic | No | Yeung et al. 2017^28^;  Tan et al. 2011^9^;  Ngeow et al. 2014^7^;  Ngeow et al. 2011^29^ | Tan: identified in cohort with relaxed CS criteria; Ngeow: identified in patients with CS/CS-like syndromes - present in secondary malignant neoplasms; 2011: identified in CS with Thyroid cancer; Yeung: identified in patient with DD with no ASD; Mighell: identified in CC cohort; different patients presented with ASD (young) and cancer (adult); also present in SFARI | Both |
| Y68D | Cancer | No | Likely pathogenic | No | Loffeld et al. 2006^30^ | Proteus syndrome and CS | CS |
| Y68H | Cancer | Yes | Pathogenic/Likely pathogenic | No | Frazier et al. 2014^1^;  Tan et al. 2011^9^;  Pilarski et al. 2011^5^;  Bilbao et al. 2006^18^;  Marsh et al. 1998^27^;  Mighell et al. 2020^3^ | Tan: identified in cohort with relaxed CS criteria; Pilarski: Study on CS and BRRS phenotypic patients; Bilbao: identified in endometrial cancer patients; Marsh: identified in BRRS families; Mighell: identified in CC cohort; patient had BRRS symptoms including developmental delay | Both |
| Y68N | PHTS1 | Yes | - | No | Klein et al. 2013^19^;  Mighell et al. 2020^3^ | identified in PHTS patient with BRRS; epilepsy | ASD |
| L70P | Cancer | No | - | No | Mighell et al. 2020^3^ | Found in CC cohort; patient developed thyroid cancer | Cancer |
| L70V | - | Yes | - | No | Hobert et al. 2014^31^ | - | ASD2 |
| D77G | - | No | - | Yes | - | - | Non-pathogenic |
| T78A | - | Yes | - | No | Schaaf et al. 2011^32^ | Present in SFARI/VariCarta | ASD2 |
| T78N | - | No | - | Yes | - | - | Non-pathogenic |
| A79T | Cancer | Yes | Likely benign | Yes | Aspromonte et al. 2019^33^;  Tan et al. 2011^9^;  Pilarski et al. 2011^5^;  Mighell et al. 2020^3^ | Tan: identified in cohort with relaxed CS criteria; Pilarski: Study on CS and BRRS phenotypic patients; Mighell: identified in CC cohort; 3 different patients exhibited ductal breast carcinoma; also present in SFARI | Both |
| N82T | Cancer | No | - | No | Figer et al. 2002^34^ | Found in breast cancer | Cancer |
| Y88C | PHTS1 | Yes | - | No | Mester et al. 2012^12^;  Mighell et al. 2020^3^ | Mester:Found in PHTS; patient had Macro; DD and hypotonia; Mighell: CC cohort patient showed variable delay; macro | ASD |
| Y88H | Cancer | No | - | No | Tan et al. 2011^9^;  Pilarski et al. 2011^5^ | Patients met relaxed criteria for CS; Study on CS and BRRS phenotypic patients | CS |
| D92A | Cancer | No | Likely pathogenic | No | Bubien et al. 2013^11^;  Mighell et al. 2020^3^ | Bubien:identified in patient with CS and endometrial cancer; Mighell: identified in CC cohort; patient had ductal breast cancer | Cancer |
| D92G | Cancer | No | Likely pathogenic | No | Bilbao et al. 2006^18^ | identified in patients with endometrial cancer | Cancer |
| D92N | - | Yes | - | No | Iossifov et al. 2014^35^;  Ji et al. 2016^36^;  Krupp et al. 2017^37^;  O’Roak et al. 2014^38^;  Lim et al. 2017^39^; | Present in SFARI/VARICARTA | ASD2 |
| H93D | PHTS1 | Yes | Uncertain significance | No | Mighell et al. 2020^3^ | Found in CC cohort; patients exhibited ASD/PHTS symptoms | ASD |
| H93R | Cancer | Yes | Pathogenic | No | Butler et al. 2005^40^;  Tan et al. 2011^9^;  Bilbao et al. 2006^18^ | Tan:identified in cohort with relaxed CS criteria; Bilbao: identified in endometrial cancer; Butler: identified in ASD patient with macrocephaly without CS; Also present in SFARI | Both |
| H93Y | Cancer | Yes | Likely pathogenic | No | Frazier et al. 2014^1^;  Tan et al. 2011^9^;  Ngeow et al. 2014^7^;  Kohno et al. 1998^41^;  Mighell et al. 2020^3^ | Tan: identified in cohort with relaxed CS criteria; Ngeow: PTEN mutations in second malignant neoplasms; Kohno: identified in CS disease; no cancer; Mighell: identified in CC cohort; different patients exhibited PHTS symptoms and ASD | Both |
| P95L | PHTS1 | No | Pathogenic | No | Hansen-Kiss et al. 2017^2^;  Tan et al. 2011^9^;  Pilarski et al. 2011^5^;  Mighell et al. 2020^3^ | HK et al: Patients tested for: PHTS; CS; BRRS; Pateint showed GI polyps; Tan et al: relaxed CS cohort; Pilarski: Study on CS and BRRS phenotypic patients; Mighell: PHTS patient | PHTS |
| P95T | PHTS1 | Yes | Likely pathogenic | No | Mighell et al. 2020^3^ | Patient in CC cohort exhibited DD; dysmorphic features; macrocephaly | ASD |
| P96Q | PHTS1 | Yes | - | No | Bussaglia et al. 2002^42^;  Mighell et al. 2020^3^ | Found in CS families; Found in CC cohort in patient with ASD; DD; Macro and BRRS symptoms | ASD |
| P96R | PHTS1 | No | Uncertain significance | No | Tan et al. 2011^9^;  Pilarski et al. 2011^5^ | Tan: Patients met relaxed criteria for CS; Study on CS and BRRS phenotypic patients; Mighell: identified in CC cohort in patient having PHTS symptoms (no cancer) | PHTS |
| Q97H | - | No | - | Yes | - | - | Non-pathogenic |
| L98R | Cancer | No | Uncertain significance | No | Mighell et al. 2020^3^ | Found in CC cohort in patient with kidney and thyroid cancer | Cancer |
| L100P | Cancer | No | Uncertain significance | No | Mighell et al. 2020^3^ | Found in CC cohort in patient with PHTS and kidney cancer | Cancer |
| I101T | Cancer | Yes | Pathogenic/Likely pathogenic | No | Ciaccio et al. 2019^13^;  Ruzzo et al. 2019^43^;  Satterstrom et al. 2020^44^;  O’Roak et al. 2014^38^;  Tan et al. 2011^9^;  Pilarski et al. 2011^5^;  Mighell et al. 2020^3^ | Tan: identified in cohort with relaxed CS criteria; Pilarski: Study on CS and BRRS phenotypic patients; Mighell: identified in patient with ASD and DD; also present in SFARI and VariCarta | Both |
| C105F | - | Yes | - | No | Yeung et al. 2018^28^ | Present in SFARI | ASD2 |
| C105Y | Cancer | No | Likely pathogenic | No | Marsh et al. 1999^15^ | identified in BRRS families | CS |
| D107G | PHTS1 | Yes | - | No | Vanderver et al. 2014^6^ | MRI scans on patients with PHTS; patient showed DD; Macro | ASD |
| D107V | PHTS2 | Yes | Pathogenic | No | O’Roak et al. 2014^38^;  Hansen-Kiss et al. 2017^2^ | Patients tested for: PHTS; CS; BRRS; Pateint showed ASD; ID; CS; Thyroid; Derm; p.f.; Also present in VariCarta | PHTS |
| L108P | Cancer | No | Pathogenic/Likely pathogenic | No | Ngeow et al. 2014^7^;  Bubien et al. 2013^11^;  Mighell et al. 2020^3^ | Ngeow: Patients having CS/CS-like syndromes; present in secondary malignant neoplasms; Bubien: identified in CS via genetic test; Mighell: identified in CC cohort patient with breast and endometrial cancer | Cancer |
| L108R | Cancer | No | - | No | Mighell et al. 2020^3^ | Found in CC cohort in patient with ductal breast carcinoma | Cancer |
| Q110E | Cancer | No | Uncertain significance | Yes | Mighell et al. 2020^3^ | Found in CC cohort in patient with breast cancer | Cancer |
| W111R | PHTS1 | Yes | Likely pathogenic | No | Zhou et al. 2001^45^;  Mighell et al. 2020^3^ | Zhou:identified in Proteus syndrome; Mighell: identified in CC cohort; patient had epilepsy and learning disabilities; dermatological macules | ASD |
| L112P | Cancer | No | Pathogenic | No | Tan et al. 2011^9^ | identified in cohort with relaxed CS criteria | CS |
| L112V | Cancer | No | - | No | Tan et al. 2011^9^ | Patients met relaxed criteria for CS | CS |
| S113I | Cancer | No | Uncertain significance | No | Whitworth et al. 2015^46^ | Found in Multiple Primary Malignant tumors | Cancer |
| D115G | Cancer | No | Conflicting interpretations of pathogenicity | No | Shirts et al. 2016^47^ | Found in patients with colorectal cancer | Cancer |
| H118P | - | Yes | - | No | Orrico et al. 2009^48^ | Present in SFARI | ASD2 |
| V119L | Cancer | No | Likely pathogenic | No | De Vivo et al. 2000^49^ | identified in women with multiple cancers (endometrial; bilaterial ovarian; invasive ductal breast; lung) | Cancer |
| A120E | Cancer | No | Likely pathogenic | No | Tan et al. 2011^9^;  Pilarski et al. 2011^5^;  Mighell et al. 2020^3^ | Tan: identified in cohort with relaxed CS criteria; Pilarski: Study on CS and BRRS phenotypic patients; Mighell: identified in patient with adenomatous polyps and prostate cancer | Cancer |
| I122S | Cancer | Yes | Uncertain significance | No | Bilbao et al. 2006^18^;  Mighell et al. 2020^3^ | Found in endometrial cancer; Found in CC cohort patient with ASD and DD | Both |
| H123D | Cancer | No | Pathogenic | No | Bussaglia et al. 2002^42^ | identified in CS families | CS |
| H123Q | Cancer | Yes | Pathogenic | No | McBride et al. 2010^50^;  Hansen-Kiss et al. 2017^2^; Kersseboom et al. 2012^51^ | Patients tested for: PHTS; CS; BRRS; Pateint showed ASD; ID; Kersseboom: identified in CS patient with colorectal cancer | Both |
| H123R | Cancer | No | Likely pathogenic | No | Bilbao et al. 2006^18^ | identified in endometrial cancer | Cancer |
| H123Y | Cancer | No | Pathogenic/Likely pathogenic | No | Myers et al. 1997^24^ | identified in endometrial cancer | Cancer |
| C124R | Cancer | Yes | Pathogenic | No | Tan et al. 2011^9^;  Bilbao et al. 2006^18^;  Marsh et al. 1998^27^;  Mighell et al. 2020^3^ | Tan:identified in cohort with relaxed CS criteria; Bilbao: identified in endometrial cancer; Marsh: identified in CS family; Mighell: identified in patient with mental retardation; leukodystrophy | Both |
| C124S | Cancer | No | Likely pathogenic | No | Tan et al. 2011^9^ | identified in cohort with relaxed CS criteria | CS |
| C124W | Cancer | No | - | No | Tan et al. 2011^9^;  Pilarski et al. 2011^5^;  Mighell et al. 2020^3^ | Patients met relaxed criteria for CS; Study on CS and BRRS phenotypic patients | CS |
| C124Y | PHTS1 | No | Likely pathogenic | No | Sawada et al. 2004^52^ | Sawada: Mutation found in 39 y/o suspected CS patient | PHTS |
| K125E | Cancer | No | - | No | Nassif et al. 2014^26^ | Found in colorectal cancer | Cancer |
| A126P | PHTS1 | No | - | No | Tan et al. 2011^9^;  Mighell et al. 2020^3^ | Patients met relaxed criteria for CS; identified in CC cohort in patient with PHTS but no cancer | PHTS |
| A126V | Cancer | Yes | Conflicting interpretations of pathogenicity | No | Bilbao et al. 2006^40^ | Found in endometrial cancer | Both |
| G127E | PHTS1 | Yes | Likely pathogenic | No | Mighell et al. 2020^3^ | Mighell: identified in CC cohort in patient with PHTS and ASD; DD; mental retardation | ASD |
| G127R | Cancer | No | Likely pathogenic | No | Whitworth et al. 2015^46^;  Lachlan et al. 2007^53^ | Whitworth: Mutation found in patient with multiple primary malignant tumours; Lachlan: mutation found in CS/CS-like patients | Cancer |
| K128E | PHTS1 | Yes | Uncertain significance | No | Tan et al. 2011^9^;  Mighell et al. 2020^3^ | Patients met relaxed criteria for CS; in patient with PHTS symptoms with DD | ASD |
| K128N | Cancer | No | Likely pathogenic | No | Ngeow et al. 2011^29^;  Lachlan et al. 2007^53^;  Nizialek et al. 2015^54^;  Mighell et al. 2020^3^ | Ngeow: mutation found in patients with CS/CS-like conditions & thyroid cancer; Lachlan: mutation found in boy with CS & some BRRS symptoms; Nizialek: identified mutation in CS/CS-like patients; Mighell: patient with colon cancer | Cancer |
| G129E | Cancer | No | Pathogenic | No | Hansen-Kiss et al. 2017^2^; Marsh et al. 1998^27^;  Myers et al. 1997^24^;  Pilarski et al. 2011^5^;  Mighell et al. 2020^3^ | HK: Patients tested for: PHTS; CS; BRRS; Pateint showed Thyroid; GI; p.f. derm; CS; BRRS; Pilarski: Study on CS and BRRS phenotypic patients; Marsh 1998: identified in CS family; Myers: found in CS; Mighell: found in PHTS patient | CS |
| G129R | Cancer | Yes | Pathogenic | No | Myers et al. 1997^24^;  Mighell et al. 2020^3^ | Myers:Identified in glioblastoma; Mighell: identified in 2 separate cases: 5 yo child exhibited epilepsy/DD; 12 yo child exhibited BRRS/PHTS | Both |
| G129V | PHTS2 | Yes | Uncertain significance | No | Pilarski et al. 2011^5^;  Mighell et al. 2020^3^ | Study on CS and BRRS phenotypic patients; in CC cohort patient exhibited ASD; thyroid cancer | PHTS |
| R130G | Cancer | No | - | No | Tan et al. 2011^9^;  Pilarski et al. 2011^5^;  Ngeow et al. 2014^7^;  Mighell et al. 2020^3^ | Patients met relaxed criteria for CS; Study on CS and BRRS phenotypic patients; PTEN mutation in secondary malignant neoplasms; CC cohort in patient with breast; thyroid cancer | Cancer |
| R130L | Cancer | No | Pathogenic/Likely pathogenic | No | Tan et al. 2011^9^;  Pilarski et al. 2011^5^;  Ngeow et al. 2014^7^;  Marsh et al. 1998^27^;  Bubein et al. 2013^11^;  Mighell et al. 2020^3^ | Tan: identified in cohort with relaxed CS criteria; Pilarski: Study on CS and BRRS phenotypic patients; Ngeow: PTEN mutation in secondary malignant neoplasms; Marsh 1998: identified in CS family; Bubien: identified in CS but no cancer reported; Mighell: identified in PHTS patient with cancer | Cancer |
| R130P | Cancer | Yes | Pathogenic | No | Ciaccio et al. 2019^13^;  Chang et al. 2016^55^ | Identified in different cancers; also present in SFARI | Both |
| R130Q | Cancer | Yes | Pathogenic | No | Ciaccio et al. 2019^13^;  Tan et al. 2011^9^;  Pilarski et al. 2011^5^;  Ngeow et al. 2014^7^;  Bubein et al. 2013^56^;  Bilbao et al. 2006^18^;  Mighell et al. 2020^3^ | Tan: identified in cohort with relaxed CS criteria; Pilarski: Study on CS and BRRS phenotypic patients; Ngeow: PTEN mutation in secondary malignant neoplasms; Bilbao: identified in endometrial cancer; Bubien: identified in CS patients with no reported cancer; Mighell: identified in different patients with cancers ranging from breast; to cervical to oesophageal; also present in SFARI | Both |
| T131I | PHTS1 | Yes | - | No | Iossifov et al. 2014^35^;  Ji et al. 2016^36^;  O’Roak et al. 2012^57^;  O’Roak et al. 2014^38^; Satterstrom et al. 2018^44^;  Lim et al. 2017^39^;  Bubien et al. 2013^11^ | Bubien: identified in CS patient with ASD but no cancer; also present in VariCarta | ASD |
| T131N | Cancer | No | - | No | Davies et al. 1999^14^ | Found in glioma tumors; acquired somatic mutation | Cancer |
| G132A | PHTS1 | Yes | - | No | Tan et al. 2007^58^ | Found in PHTS patient with Macro and speech delay | ASD |
| G132D | Cancer | Yes | Likely pathogenic | No | Frazier et al. 2014^1^;  Tan et al. 2011^9^;  Bubien et al. 2013^11^;  Mighell et al. 2020^3^ | Tan: identified in cohort with relaxed CS criteria; Bubien: identified in CS with and without breast cancer; Mighell: found in 2 patients; one had ASD/DD and other had different cancers | Both |
| G132V | Cancer | No | Pathogenic/Likely pathogenic | No | Tan et al. 2011^9^;  Pilarski et al. 2011^5^ | Tan: identified in cohort with relaxed CS criteria; Pilarski: Study on CS and BRRS phenotypic patients | CS |
| M134I | PHTS1 | Yes | Likely pathogenic | No | Busa et al. 2013^59^ | Busa: found in patients with macrocephaly; hypotonia & learning disabilities without other PHTS symptoms or malignant tumours; | ASD |
| M134L | - | Yes | - | No | De Rubeis et al. 2014^60^ | Present in SFARI | ASD2 |
| M134R | Cancer | No | Likely pathogenic | No | Figer et al. 2002^34^;  Mighell et al. 2020^3^ | Figer: identified in patient with BRRS ; Mighell: identified in patient with PHTS and cancers | Cancer |
| M134T | Cancer | Yes | Pathogenic | No | McBride et al. 2010^50^;  Hansen-Kiss et al. 2017^2^; Busch et al. 2013^61^;  Mighell et al. 2020^3^ | Patients tested for: PHTS; CS; BRRS; Pateint showed ASD; ID; Derm; McBride: patient showed ASD-M; PHTS &BRR syndrome; Busch: identified in patients with PHTS; having breast and thyroid cancer; Mighell: patient showed macrocephaly; also present in SFARI | Both |
| I135K | Cancer | No | Likely pathogenic | No | Tan et al. 2011^9^;  Pilarski et al. 2011^5^ | Tan: identified in cohort with relaxed CS criteria; Pilarski: Study on CS and BRRS phenotypic patients | CS |
| I135R | Cancer | Yes | - | No | Bilbao et al. 2006^18^;  Boccone et al. 2006^62^ | Found in endometrial cancer; found in patient with BRRS/ASD | Both |
| I135V | PHTS2 | Yes | Pathogenic/Likely pathogenic | No | Bubien et al. 2013^11^;  Marsh et al. 1999^15^;  Caux et al. 2007^63^ | Marsh: identified in BRRS family; Bubien: identified in CS patient with retardation/ASD; Caux: identified in CS | PHTS |
| C136R | PHTS2 | Yes | Pathogenic | No | Bubien et al. 2013^11^;  Tan et al. 2011^9^;  Pilarski et al. 2011^5^;  Ngeow et al. 2014^7^;  Bilbao et al. 2006^18^;  Mighell et al. 2020^3^ | Tan: identified in cohort with relaxed CS criteria; Pilarski: Study on CS and BRRS phenotypic patients; Ngeow: PTEN mutation in secondary malignant neoplasms; Bilbao: identified in endomterial cancer; Bubien et al: identified in CS patients with and without breast cancer and retardation and asd; Mighell: identified in different patient exhibiting different cancers; ASD/DD | PHTS |
| C136W | PHTS1 | No | Likely pathogenic | No | Shirts et al. 2016^47^ | Suspected CS; no cancer | PHTS |
| C136Y | Cancer | No | Pathogenic | No | Tan et al. 2011^9^;  Pilarski et al. 2011^5^;  Ngeow et al. 2011^29^;  Mighell et al. 2020^3^ | Tan: identified in cohort with relaxed CS criteria; Pilarski: Study on CS and BRRS phenotypic patients; Ngeow: identified in CS with thyroid cancer; Mighell: identified in different patients exhibiting PHTS symptoms | Cancer |
| L139F | PHTS1 | Yes | - | No | Mighell et al. 2020^3^ | Found in CC cohort in patient with ASD; DD; Macro and BRRS symptoms | ASD |
| L139I | - | No | - | Yes | - | - | Non-pathogenic |
| H141R | Cancer | Yes | Uncertain significance | No | Mighell et al. 2020^3^ | Found in CC cohort in separate patients exhibiting epilepsy/ASD (7 yo) and cancer (52 yo) | Both |
| R142P | PHTS1 | No | Uncertain significance | No | Bubien et al. 2013^11^ | Found in CS Patient with no cancer | PHTS |
| R142W | Cancer | Yes | Uncertain significance | Yes | Bilbao et al. 2006^18^;  Mighell et al. 2020^3^ | Found in endometrial cancer; Found in CC cohort patient with variable delay/epilepsy ( 7 yo) and learning disabilities (36 yo) | Both |
| G143D | Cancer | No | Uncertain significance | No | Bilbao et al. 2006^18^ | Found in endometrial cancer | Cancer |
| E150G | - | Yes | Uncertain significance | No | Satterstrom et al. 2020^44^ | Present in SFARI/VariCarta | ASD2 |
| E150Q | Cancer | No | - | No | Nassif et al. 2004^26^ | Found in colorectal cancer | Cancer |
| A151D | PHTS1 | No | - | No | Bubien et al. 2013^11^ | Found in CS Patient with no cancer | PHTS |
| L152P | Cancer | No | Uncertain significance | No | Tan et al. 2011^9^;  Pilarski et al. 2011^5^ | Patients met relaxed criteria for CS; Study on CS and BRRS phenotypic patients | CS |
| D153N | Cancer | No | Uncertain significance | No | Nassif et al. 2004^26^ | Found in colorectal cancer | Cancer |
| D153Y | Cancer | No | - | No | Nassif et al. 2004^26^ | Found in colorectal cancer | Cancer |
| F154S | - | No | - | Yes | - | - | Non-pathogenic |
| Y155C | Cancer | Yes | Pathogenic | No | Tan et al. 2011^9^;  Bilbao et al. 2006^18^;  Bubien et al. 2013^11^;  Mighell et al. 2020^3^ | Tan:identified in cohort with relaxed CS criteria; Bilbao: identified in endometrial cancer; Bubien: identified in CS patient with breast cancer; Mighell:observed in different patients exhibiting PHTS; Cancer and ASD | Both |
| Y155H | PHTS1 | Yes | Pathogenic | No | Tan et al. 2011^9^;  Pilarski et al. 2011^5^;  Mighell et al. 2020^3^ | Tan: identified in cohort with relaxed CS criteria; Pilarski: Study on CS and BRRS phenotypic patients; Mighell: observed in patient with PHTS including benign tumors; mental retardation and epilepsy | ASD |
| Y155N | Cancer | Yes | - | No | Bubien et al. 2013^11^;  Mighell et al. 2020^3^ | Found in CS Patient with melanoma; found in CC cohort patients with ASD/DD and BRRS; and BRRS with testicular cancer | Both |
| E157G | - | Yes | Conflicting interpretations of pathogenicity | Yes | Varga et al. 2009^21^ | - | ASD2 |
| E157Q | PHTS1 | Yes | - | No | Hansen-Kiss et al. 2017^2^ | Patients tested for: PHTS; CS; BRRS; Pateint showed ASD; DD | ASD |
| V158L | Cancer | No | - | No | De Vivo et al. 2000^49^ | Found in women with multiple cancers: lymphoma; lower genital tract and invasive ductal breast | Cancer |
| R159G | Cancer | No | Likely pathogenic | No | Tan et al. 2011^9^;  Mighell et al. 2020^3^ | Tan: identified in cohort with relaxed CS criteria; Mighell: found in intraductal cancer of the breast | Cancer |
| R159T | Cancer | No | - | No | Tan et al. 2011^9^;  Mighell et al. 2020^3^ | Patients met relaxed criteria for CS; in CC patient with PHTS and intraductal carcinoma | Cancer |
| D162E | Cancer | Yes | Likely pathogenic | No | Tan et al. 2011^9^;  Mighell et al. 2020^3^;  Shirts et al. 2016^47^ | Tan:identified in cohort with relaxed CS criteria; Shirts: identified in patients with breast cancer and lymphoma; Mighell: identified in CC cohort patient with DD/BRRS features | Both |
| K164N | - | Yes | - | Yes | Yeung et al. 2017^64^ | Present in SFARI | ASD2 |
| G165E | Cancer | No | Uncertain significance | No | Nelen et al. 1999^65^ | Found in CS patient with thyroid cancer | Cancer |
| G165R | Cancer | No | Pathogenic | No | Tan et al. 2011^9^;  Pilarski et al. 2011^5^;  Myers et al. 1997{Myers, 1997 #24};  Mighell et al. 2020^3^ | Tan: identified in cohort with relaxed CS criteria; Pilarski: Study on CS and BRRS phenotypic patients; Myers: found in glioblastoma; Mighell: found in patient with Lhermitte-Duclos disease | Cancer |
| G165V | PHTS1 | Yes | - | No | Tammimies et al. 2015^66^;  Marsh et al. 1998^27^;  Mighell et al. 2020^3^ | Marsh:identified in familial CS; Mighell: identified in ASD/DD patient; also present in SFARI | ASD |
| T167A | - | No | - | Yes | - | - | Non-pathogenic |
| T167N | - | Yes | - | No | Iossifov et al. 2014^35^;  Ji et al. 2016^36^;  Krupp et al. 2017^37^;  O’Roak et al. 2012^57^;  O’Roak et al. 2014^38^; Satterstrom et al. 2018^44^;  Lim et al. 2017^39^ | Present in SFARI/VariCarta | ASD2 |
| T167P | Cancer | No | - | No | Myers et al. 1997^24^ | Found in breast cancer | Cancer |
| S170I | PHTS2 | Yes | Pathogenic | No | Tan et al. 2011^9^;  Pilarski et al. 2011^5^;  Ngeow et al. 2014^7^;  Ngeow et al. 2011^29^;  Mighell et al. 2020^3^ | Tan: identified in cohort with relaxed CS criteria; Pilarski: Study on CS and BRRS phenotypic patients; Ngeow: PTEN mutation in secondary malignant neoplasms; Ngeow 2011: identified in CS with thyroid cancer; Mighell: observed in patient with PHTS; having ASD; endometrial and thyroid cancer | PHTS |
| S170R | Cancer | Yes | Pathogenic | No | Ciaccio et al. 2019^13^;  Tan et al. 2011^9^;  Pilarski et al. 2011^5^;  Marsh et al. 1998^27^ & 1999^15^; Myers et al. 1997^24^;  Bubien et al. 2013^11^ | Tan: identified in cohort with relaxed CS criteria; Pilarski: Study on CS and BRRS phenotypic patients; Marsh et al 1998& 1999: identified in BRRS families; Myers: BRRS patient; Bubien et al: identified in CS patient with and without breast cancer, also present in SFARI | Both |
| S170T | - | Yes | - | No | Yeung et al. 2018^28^ | Present in SFARI | ASD2 |
| Q171E | PHTS1 | Yes | Uncertain significance | No | Vanderver et al. 2014^6^ | MRI screens in patients with PHTS; patient observed to have DD; ASD | ASD |
| R173C | PHTS2 | Yes | Pathogenic | No | Hansen-Kiss et al. 2017^2^;  Tan et al. 2011^9^;  Ngeow et al. 2014^7^;  Bilbao et al. 2006^18^;  Mighell et al. 2020^3^ | HK et al: Patients tested for: PHTS; CS; BRRS; Pateint showed ID; Tan et al: relaxed CS cohort; Ngeow et al: PTEN mutations in secondary malignant neoplasms; Bilbao: identified in endometrial cancer; Mighell: identified in different patients exhibiting ASD/DD; BRRS symptoms and different malignancies | PHTS |
| R173H | PHTS2 | Yes | Pathogenic/Likely pathogenic | Yes | McBride et al. 2010^50^;  Bubein et al. 2013^11^;  Varga et al. 2009^21^;  Hansen-Kiss et al. 2017^2^; Davies et al. 1999^14^;  Bilbao et al. 2006^18^ | HK:Patients tested for: PHTS; CS; BRRS; Pateint showed ASD; ID; Thyroid; Derm; Davies: identified in gliomas - acquired somatic mutation; Bilbao: identified in endometrial cancer; Bubien et al: identified in CS patient with retardation/ASD; Also present in SFARI | PHTS |
| R173P | PHTS1 | No | Likely pathogenic | No | Kirches et al. 2010^67^ | Mutation found in patient with Lhermitte-Duclos disease and psychosis | PHTS |
| Y174C | Cancer | No | Likely pathogenic | No | Wang et al. 2010^68^ | mutation found in metatastic melanoma excised from patient postmortem. Determined not to be caused by UV radiation | Cancer |
| Y174N | Cancer | No | Likely pathogenic | No | LaDuca et al. 2014^69^ | identified in cancer cohort | Cancer |
| Y176C | PHTS1 | Yes | Uncertain significance | Yes | Orrico et al. 2009^48^;  Tan et al. 2011^9^;  Pilarski et al. 2011^5^ | Tan: identified in relaxed CS criteria; Pilarski: identified in CS/BRRS families; also present in SFARI | ASD |
| L181P | PHTS1 | No | Conflicting interpretations of pathogenicity | No | Tan et al. 2011^9^;  Pilarski et al. 2011^5^;  Mighell et al. 2020^3^ | Patients met relaxed criteria for CS; Study on CS and BRRS phenotypic patients; CC cohort patient exhibited PHTS symptoms with benign polyps | PHTS |
| L182S | PHTS2 | Yes | Uncertain significance | No | Tan et al. 2011^9^;  Pilarski et al. 2011^5^ | Tan: identified in relaxed CS criteria; Pilarski: identified in CS/BRRS families | PHTS |
| Y188H | PHTS1 | Yes | - | No | Tan et al. 2011^9^;  Mighell et al. 2020^3^ | Patients met relaxed criteria for CS; CC cohort patients exhibited macrocephaly; with/without ASD | ASD |
| M198I | - | No | - | Yes | - | - | Non-pathogenic |
| F200S | PHTS1 | Yes | Uncertain significance | No | Pilarski et al. 2011^5^;  Mighell et al. 2020^3^ | Study on CS and BRRS phenotypic patients; in CC cohort patient exhibited epilepsy/DD; pappilomas | ASD |
| T202I | PHTS1 | Yes | Pathogenic | No | Varga et al. 2009^21^;  Hansen-Kiss et al. 2017^2^ | Patients tested for: PHTS; CS; BRRS; Pateint showed ID; Derm; also present in SFARI | ASD |
| M205V | Cancer | No | Uncertain significance | Yes | Tan et al. 2011^9^;  Mighell et al. 2020^3^ | Patients met relaxed criteria for CS;Found in CC cohort in PHTS patient with cancer | Cancer |
| F206L | - | Yes | - | No | Schaaf et al. 2011^32^ | Present in SFARI/VariCarta | ASD2 |
| C211W | PHTS1 | Yes | Conflicting interpretations of pathogenicity | No | Vanderver et al. 2014^6^ | MRI screens in patients with PHTS; patient observed to have DD; ASD; Macro | ASD |
| V217A | Cancer | No | - | No | Nassif et al. 2004^26^ | Found in colorectal cancer | Cancer |
| V217D | Cancer | No | - | No | Kim et al. 2005^70^ | Found in CS patient | CS |
| V217I | Cancer | No | Pathogenic | No | Celebi et al. 2000^10^ | Mutation found in melanoma cells excised from patient | Cancer |
| L220V | PHTS1 | No | - | No | Mighell et al. 2020^3^ | Found in CC cohort in PHTS patient with dysmorphic features and generalized overgrowth | PHTS |
| K221R | - | No | - | Yes | - | - | Non-pathogenic |
| I224K | Cancer | No | - | No | Mighell et al. 2020^3^ | Found in CC cohort with breast cancer | Cancer |
| S229L | - | No | - | Yes | - | - | Non-pathogenic |
| R234Q | Cancer | No | Uncertain significance | No | Staal et al. 2002^71^ | Found in brain tumours | Cancer |
| M239R | PHTS2 | Yes | Uncertain significance | No | Mighell et al. 2020^3^ | Found in CC cohort in PHTS with BRRS; DD and cancer | PHTS |
| M239T | PHTS1 | Yes | Uncertain significance | Yes | Mighell et al. 2020^3^ | Found in CC cohort in patient with Learning disabilities | ASD |
| F241L | PHTS1 | Yes | Likely pathogenic | No | Yuen et al. 2017^72^ | Identified in patient with macrocephaly in family with ASD history; also present in VariCarta | ASD |
| F241S | Cancer | Yes | Likely pathogenic | No | Butler et al. 2005^40^;  Tan et al. 2011^9^ | identified in cohort with relaxed CS criteria; Butler et al identified protein change identified in boy with ASD & macrocephaly; also present in SFARI | Both |
| P246L | PHTS1 | Yes | Pathogenic | No | Yuen et al. 2017^72^;  Du et al. 2018^73^;  Epi4K Consortium et al. 2013^74^;  Vanderver et al. 2014^6^;  Tan et al. 2011^9^;  Marsh et al. 1999^15^;  Mighell et al. 2020^3^ | Tan:identified in cohort with relaxed CS criteria; Vanderver: MRI in patients with PHTS; patient showed DD; Macro and ASD; Marsh 1999: identified in BRRS; Mighell: identified in PHTS with DD; also present in SFARI | ASD |
| L247S | PHTS1 | Yes | Likely pathogenic | No | Negishi et al. 2017^75^ | Negishi identified this mutation in a young girl with megalencephaly/developmental delays; also present in SFARI | ASD |
| P248R | - | No | - | Yes | - | - | Non-pathogenic |
| G251V | Cancer | No | Likely pathogenic | No | Mighell et al. 2020^3^ | Found in CC cohort in patient with cancer | Cancer |
| D252G | PHTS1 | Yes | Likely pathogenic | No | Butler et al. 2005^40^;  Tan et al. 2011^9^;  Pilarski et al. 2011^5^;  Bubien et al. 2013^11^ | Tan: identified in cohort with relaxed CS criteria; Pilarski: Study on CS and BRRS phenotypic patients; Bubien: identified in CS with no cancer; Butler: identified in ASD patient with macrocephaly but no CS; also present in SFARI | ASD |
| D252V | PHTS1 | Yes | Uncertain significance | No | Frazier et al. 2015^1^;  Mighell et al. 2020^3^ | identified in PHTS patient with ASD/DD | ASD |
| K254T | Cancer | Yes | Likely pathogenic | No | Ngeow et al. 2011^29^;  Tan et al. 2011^9^;  Mighell et al. 2020^3^ | Ngeow reports this mutation in multiple people with CD; Tan et al: identified in cohorts with relaxed CS criteria; Ngeow et al 2011: CS patients with thyroid cancer; Mighell: identified in PHTS in different patients; one with DD | Both |
| V255A | - | Yes | - | No | Klein et al. 2013^19^ | - | ASD2 |
| K260R | Cancer | No | Uncertain significance | Yes | Pilarski et al. 2011^5^ | Study on CS and BRRS phenotypic patients | CS |
| N262S | - | No | - | Yes | - | - | Non-pathogenic |
| L265R | - | No | - | Yes | - | - | Non-pathogenic |
| K267N | - | Yes | Uncertain significance | No | Ciaccio et al. 2019^13^ | S | ASD2 |
| D268E | No | No | Likely benign | Yes | - | - | Non-pathogenic |
| M270K | - | Yes | - | No | Wu et al. 2020^76^ | Present in SFARI | ASD2 |
| H272P | Cancer | No | Likely pathogenic | No | Mighell et al. 2020^3^ | Identified in PHTS patient with melanoma | Cancer |
| W274L | PHTS1 | Yes | Pathogenic | No | McBride et al. 2010^50^;  Hansen-Kiss et al. 2017^2^ | Patients tested for: PHTS; CS; BRRS; Pateint showed ID; also present in SFARI | ASD |
| N276S | - | Yes | - | No | Orrico et al. 2009^48^ | Present in SFARI | ASD2 |
| T277I | PHTS1 | Yes | Uncertain significance | No | Busa et al. 2013^59^ | Found in young patient with DD and macrocephaly | ASD |
| T277R | PHTS2 | Yes | Likely pathogenic | No | Bubien et al. 2013^11^;  Banneau et al. 2010^77^ | Bubien: identified in CS with retardation and ASD; and vocal cord cancer; Banneau: identified in breast cancer | PHTS |
| P281A | PHTS1 | No | Uncertain significance | No | Mighell et al. 2020^3^ | Found in CC cohort in patient with PHTS | PHTS |
| K289E | PHTS1 | No | Conflicting interpretations of pathogenicity | Yes | Chi et al. 1998^78^ | Found in CS GI polyps | PHTS |
| Q298E | Cancer | No | Uncertain significance | Yes | Mighell et al. 2020^3^ | Found in CC cohort in patient with cancer | Cancer |
| S302G | - | No | - | Yes | - | - | Non-pathogenic |
| T319I | - | No | - | Yes | - | - | Non-pathogenic |
| L320S | PHTS1 | Yes | - | No | Ueno et al. 2019^79^;  Mighell et al. 2020^3^ | Mighell:identified in ASD patient; also present in SFARI | ASD |
| N323K | Cancer | No | - | No | Nassif et al. 2004^26^ | Found in colorectal cancer | Cancer |
| D324Y | - | No | - | Yes | - | - | Non-pathogenic |
| D326N | - | Yes | - | No | Buxbaum et al. 2007^8^ | Present in SFARI | ASD2 |
| R335L | Cancer | No | Uncertain significance | No | Sawada et al. 2000^80^ | Found in CS patient | CS |
| R335Q | PHTS1 | Yes | Pathogenic | No | Hansen-Kiss et al. 2017^2^ | Patients tested for: PHTS; CS; BRRS; Pateint showed ID; Derm | ASD |
| F337S | Cancer | No | - | No | Lachlan et al. 2007^53^ | Found in patients with CS/BRRS | CS |
| K342N | PHTS1 | No | Uncertain significance | No | Bubien et al. 2013^11^ | Found in CS patient with no cancer | PHTS |
| L345P | Cancer | No | Conflicting interpretations of pathogenicity | No | Pilarski et al. 2011^5^ | Study on CS and BRRS phenotypic patients | CS |
| L345V | Cancer | No | Uncertain significance | No | Tan et al. 2011^9^;  Pilarski et al. 2011^5^;  Ngeow et al. 2014^7^;  Mighell et al. 2020^3^ | Patients met relaxed criteria for CS; Study on CS and BRRS phenotypic patients; PTEN mutation in secondary malignant neoplasms; identified in CC cohort patient with different cancers | Cancer |
| F347L | Cancer | No | - | No | Pilarski et al. 2011^5^ | Study on CS and BRRS phenotypic patients | CS |

**References:**

1. Frazier, T. W.; Embacher, R.; Tilot, A. K.; Koenig, K.; Mester, J.; Eng, C., Molecular and phenotypic abnormalities in individuals with germline heterozygous PTEN mutations and autism. *Molecular Psychiatry* **2015,** *20* (9), 1132-1138.

2. Hansen-Kiss, E.; Beinkampen, S.; Adler, B.; Frazier, T.; Prior, T.; Erdman, S.; Eng, C.; Herman, G., A retrospective chart review of the features of PTEN hamartoma tumour syndrome in children. *J Med Genet* **2017,** *54* (7), 471-478.

3. Mighell, T. L.; Thacker, S.; Fombonne, E.; Eng, C.; O'Roak, B. J., An Integrated Deep-Mutational-Scanning Approach Provides Clinical Insights on PTEN Genotype-Phenotype Relationships. *Am J Hum Genet* **2020,** *106* (6), 818-829.

4. Lionel, A. C.; Costain, G.; Monfared, N.; Walker, S.; Reuter, M. S.; Hosseini, S. M.; Thiruvahindrapuram, B.; Merico, D.; Jobling, R.; Nalpathamkalam, T.; Pellecchia, G.; Sung, W. W. L.; Wang, Z.; Bikangaga, P.; Boelman, C.; Carter, M. T.; Cordeiro, D.; Cytrynbaum, C.; Dell, S. D.; Dhir, P.; Dowling, J. J.; Heon, E.; Hewson, S.; Hiraki, L.; Inbar-Feigenberg, M.; Klatt, R.; Kronick, J.; Laxer, R. M.; Licht, C.; MacDonald, H.; Mercimek-Andrews, S.; Mendoza-Londono, R.; Piscione, T.; Schneider, R.; Schulze, A.; Silverman, E.; Siriwardena, K.; Snead, O. C.; Sondheimer, N.; Sutherland, J.; Vincent, A.; Wasserman, J. D.; Weksberg, R.; Shuman, C.; Carew, C.; Szego, M. J.; Hayeems, R. Z.; Basran, R.; Stavropoulos, D. J.; Ray, P. N.; Bowdin, S.; Meyn, M. S.; Cohn, R. D.; Scherer, S. W.; Marshall, C. R., Improved diagnostic yield compared with targeted gene sequencing panels suggests a role for whole-genome sequencing as a first-tier genetic test. *Genet Med* **2018,** *20* (4), 435-443.

5. Pilarski, R.; Stephens, J. A.; Noss, R.; Fisher, J. L.; Prior, T. W., Predicting PTEN mutations: an evaluation of Cowden syndrome and Bannayan–Riley–Ruvalcaba syndrome clinical features. *Journal of Medical Genetics* **2011,** *48* (8), 505.

6. Vanderver, A.; Tonduti, D.; Kahn, I.; Schmidt, J.; Medne, L.; Vento, J.; Chapman, K. A.; Lanpher, B.; Pearl, P.; Gropman, A.; Lourenco, C.; Bamforth, J.-S.; Sharpe, C.; Pineda, M.; Schallner, J.; Bodamer, O.; Orcesi, S.; Oberstein, S. A. J. L.; Sistermans, E. A.; Yntema, H. G.; Bonnemann, C.; Waldman, A. T.; van der Knaap, M. S., Characteristic brain magnetic resonance imaging pattern in patients with macrocephaly and PTEN mutations. *American Journal of Medical Genetics Part A* **2014,** *164* (3), 627-633.

7. Ngeow, J.; Stanuch, K.; Mester, J. L.; Barnholtz-Sloan, J. S.; Eng, C., Second malignant neoplasms in patients with Cowden syndrome with underlying germline PTEN mutations. *J Clin Oncol* **2014,** *32* (17), 1818-24.

8. Buxbaum, J. D.; Cai, G.; Chaste, P.; Nygren, G.; Goldsmith, J.; Reichert, J.; Anckarsäter, H.; Rastam, M.; Smith, C. J.; Silverman, J. M.; Hollander, E.; Leboyer, M.; Gillberg, C.; Verloes, A.; Betancur, C., Mutation screening of the PTEN gene in patients with autism spectrum disorders and macrocephaly. *Am J Med Genet B Neuropsychiatr Genet* **2007,** *144B* (4), 484-491.

9. Tan, M. H.; Mester, J.; Peterson, C.; Yang, Y.; Chen, J. L.; Rybicki, L. A.; Milas, K.; Pederson, H.; Remzi, B.; Orloff, M. S.; Eng, C., A clinical scoring system for selection of patients for PTEN mutation testing is proposed on the basis of a prospective study of 3042 probands. *Am J Hum Genet* **2011,** *88* (1), 42-56.

10. Çelebi, J. T.; Ping, X. L.; Zhang, H.; Remington, T.; Sulica, V. I.; Tsou, H. C.; Peacocke, M., Germline PTEN mutations in three families with Cowden syndrome. *Experimental Dermatology* **2000,** *9* (2), 152-156.

11. Bubien, V.; Bonnet, F.; Brouste, V.; Hoppe, S.; Barouk-Simonet, E.; David, A.; Edery, P.; Bottani, A.; Layet, V.; Caron, O.; Gilbert-Dussardier, B.; Delnatte, C.; Dugast, C.; Fricker, J.-P.; Bonneau, D.; Sevenet, N.; Longy, M.; Caux, F., High cumulative risks of cancer in patients with &lt;em&gt;PTEN&lt;/em&gt; hamartoma tumour syndrome. *Journal of Medical Genetics* **2013,** *50* (4), 255.

12. Mester, J.; Eng, C., Estimate of de novo mutation frequency in probands with PTEN hamartoma tumor syndrome. *Genetics in Medicine* **2012,** *14* (9), 819-822.

13. Ciaccio, C.; Saletti, V.; D'Arrigo, S.; Esposito, S.; Alfei, E.; Moroni, I.; Tonduti, D.; Chiapparini, L.; Pantaleoni, C.; Milani, D., Clinical spectrum of PTEN mutation in pediatric patients. A bicenter experience. *Eur J Med Genet* **2019,** *62* (12), 103596.

14. Davies, M. P.; Gibbs, F. E.; Halliwell, N.; Joyce, K. A.; Roebuck, M. M.; Rossi, M. L.; Salisbury, J.; Sibson, D. R.; Tacconi, L.; Walker, C., Mutation in the PTEN/MMAC1 gene in archival low grade and high grade gliomas. *Br J Cancer* **1999,** *79* (9-10), 1542-8.

15. Marsh, D. J.; Kum, J. B.; Lunetta, K. L.; Bennett, M. J.; Gorlin, R. J.; Ahmed, S. F.; Bodurtha, J.; Crowe, C.; Curtis, M. A.; Dasouki, M.; Dunn, T.; Feit, H.; Geraghty, M. T.; Graham, J. M., Jr.; Hodgson, S. V.; Hunter, A.; Korf, B. R.; Manchester, D.; Miesfeldt, S.; Murday, V. A.; Nathanson, K. L.; Parisi, M.; Pober, B.; Romano, C.; Eng, C.; et al., PTEN mutation spectrum and genotype-phenotype correlations in Bannayan-Riley-Ruvalcaba syndrome suggest a single entity with Cowden syndrome. *Hum Mol Genet* **1999,** *8* (8), 1461-72.

16. Monies, D.; Abouelhoda, M.; Assoum, M.; Moghrabi, N.; Rafiullah, R.; Almontashiri, N.; Alowain, M.; Alzaidan, H.; Alsayed, M.; Subhani, S.; Cupler, E.; Faden, M.; Alhashem, A.; Qari, A.; Chedrawi, A.; Aldhalaan, H.; Kurdi, W.; Khan, S.; Rahbeeni, Z.; Alotaibi, M.; Goljan, E.; Elbardisy, H.; ElKalioby, M.; Shah, Z.; Alruwaili, H.; Jaafar, A.; Albar, R.; Akilan, A.; Tayeb, H.; Tahir, A.; Fawzy, M.; Nasr, M.; Makki, S.; Alfaifi, A.; Akleh, H.; Yamani, S.; Bubshait, D.; Mahnashi, M.; Basha, T.; Alsagheir, A.; Abu Khaled, M.; Alsaleem, K.; Almugbel, M.; Badawi, M.; Bashiri, F.; Bohlega, S.; Sulaiman, R.; Tous, E.; Ahmed, S.; Algoufi, T.; Al-Mousa, H.; Alaki, E.; Alhumaidi, S.; Alghamdi, H.; Alghamdi, M.; Sahly, A.; Nahrir, S.; Al-Ahmari, A.; Alkuraya, H.; Almehaidib, A.; Abanemai, M.; Alsohaibaini, F.; Alsaud, B.; Arnaout, R.; Abdel-Salam, G. M. H.; Aldhekri, H.; AlKhater, S.; Alqadi, K.; Alsabban, E.; Alshareef, T.; Awartani, K.; Banjar, H.; Alsahan, N.; Abosoudah, I.; Alashwal, A.; Aldekhail, W.; Alhajjar, S.; Al-Mayouf, S.; Alsemari, A.; Alshuaibi, W.; Altala, S.; Altalhi, A.; Baz, S.; Hamad, M.; Abalkhail, T.; Alenazi, B.; Alkaff, A.; Almohareb, F.; Al Mutairi, F.; Alsaleh, M.; Alsonbul, A.; Alzelaye, S.; Bahzad, S.; Manee, A. B.; Jarrad, O.; Meriki, N.; Albeirouti, B.; Alqasmi, A.; AlBalwi, M.; Makhseed, N.; Hassan, S.; Salih, I.; Salih, M. A.; Shaheen, M.; Sermin, S.; Shahrukh, S.; Hashmi, S.; Shawli, A.; Tajuddin, A.; Tamim, A.; Alnahari, A.; Ghemlas, I.; Hussein, M.; Wali, S.; Murad, H.; Meyer, B. F.; Alkuraya, F. S., Lessons Learned from Large-Scale, First-Tier Clinical Exome Sequencing in a Highly Consanguineous Population. *Am J Hum Genet* **2019,** *104* (6), 1182-1201.

17. Hagelstrom, R. T.; Ford, J.; Reiser, G. M.; Nelson, M.; Pickering, D. L.; Althof, P. A.; Sanger, W. G.; Coccia, P. F., Breast Cancer and Non-Hodgkin Lymphoma in a Young Male with Cowden Syndrome. *Pediatr Blood Cancer* **2016,** *63* (3), 544-6.

18. Bilbao, C.; Rodríguez, G.; Ramírez, R.; Falcón, O.; León, L.; Chirino, R.; Rivero, J. F.; Falcón, O., Jr.; Díaz-Chico, B. N.; Díaz-Chico, J. C.; Perucho, M., The relationship between microsatellite instability and PTEN gene mutations in endometrial cancer. *Int J Cancer* **2006,** *119* (3), 563-70.

19. Klein, S.; Sharifi-Hannauer, P.; Martinez-Agosto, J. A., Macrocephaly as a clinical indicator of genetic subtypes in autism. *Autism Res* **2013,** *6* (1), 51-6.

20. Tate, G.; Suzuki, T.; Endo, Y.; Mitsuya, T., A novel mutation of the PTEN gene in a Japanese patient with Cowden syndrome and bilateral breast cancer. *Cancer Genetics and Cytogenetics* **2008,** *184* (1), 67-71.

21. Varga, E. A.; Pastore, M.; Prior, T.; Herman, G. E.; McBride, K. L., The prevalence of PTEN mutations in a clinical pediatric cohort with autism spectrum disorders, developmental delay, and macrocephaly. *Genet Med* **2009,** *11* (2), 111-7.

22. Marsh, D. J.; Theodosopoulos, G.; Howell, V.; Richardson, A. L.; Benn, D. E.; Proos, A. L.; Eng, C.; Robinson, B. G., Rapid mutation scanning of genes associated with familial cancer syndromes using denaturing high-performance liquid chromatography. *Neoplasia* **2001,** *3* (3), 236-44.

23. Saskin, A.; Fulginiti, V.; Birch, A. H.; Trakadis, Y., Prevalence of four Mendelian disorders associated with autism in 2392 affected families. *J Hum Genet* **2017,** *62* (6), 657-659.

24. Myers, M. P.; Stolarov, J. P.; Eng, C.; Li, J.; Wang, S. I.; Wigler, M. H.; Parsons, R.; Tonks, N. K., P-TEN, the tumor suppressor from human chromosome 10q23, is a dual-specificity phosphatase. *Proc Natl Acad Sci U S A* **1997,** *94* (17), 9052-7.

25. Reardon, W.; Zhou, X. P.; Eng, C., A novel germline mutation of the PTEN gene in a patient with macrocephaly, ventricular dilatation, and features of VATER association. *J Med Genet* **2001,** *38* (12), 820-3.

26. Nassif, N. T.; Lobo, G. P.; Wu, X.; Henderson, C. J. A.; Morrison, C. D.; Eng, C.; Jalaludin, B.; Segelov, E., PTEN mutations are common in sporadic microsatellite stable colorectal cancer. *Oncogene* **2004,** *23*, 617-628.

27. Marsh, D. J.; Coulon, V.; Lunetta, K. L.; Rocca-Serra, P.; Dahia, P. L. M.; Zheng, Z.; Liaw, D.; Caron, S.; Duboué, B.; Lin, A. Y.; Richardson, A.-L.; Bonnetblanc, J.-M.; Bressieux, J.-M.; Cabarrot-Moreau, A.; Chompret, A.; Demange, L.; Eeles, R. A.; Yahanda, A. M.; Fearon, E. R.; Fricker, J.-P.; Gorlin, R. J.; Hodgson, S. V.; Huson, S.; Lacombe, D.; LePrat, F.; Odent, S.; Toulouse, C.; Olopade, O. I.; Sobol, H.; Tishler, S.; Woods, C. G.; Robinson, B. G.; Weber, H. C.; Parsons, R.; Peacocke, M.; Longy, M.; Eng, C., Mutation Spectrum and Genotype-Phenotype Analyses in Cowden Disease and Bannayan-Zonana Syndrome, Two Hamartoma Syndromes With Germline PTEN Mutation. *Human Molecular Genetics* **1998,** *7* (3), 507-515.

28. Yeung, K. S.; Tso, W. W. Y.; Ip, J. J. K.; Mak, C. C. Y.; Leung, G. K. C.; Tsang, M. H. Y.; Ying, D.; Pei, S. L. C.; Lee, S. L.; Yang, W.; Chung, B. H., Identification of mutations in the PI3K-AKT-mTOR signalling pathway in patients with macrocephaly and developmental delay and/or autism. *Mol Autism* **2017,** *8*, 66.

29. Ngeow, J.; Mester, J.; Rybicki, L. A.; Ni, Y.; Milas, M.; Eng, C., Incidence and clinical characteristics of thyroid cancer in prospective series of individuals with Cowden and Cowden-like syndrome characterized by germline PTEN, SDH, or KLLN alterations. *J Clin Endocrinol Metab* **2011,** *96* (12), E2063-71.

30. Loffeld, A.; McLellan, N. J.; Cole, T.; Payne, S. J.; Fricker, D.; Moss, C., Epidermal naevus in Proteus syndrome showing loss of heterozygosity for an inherited PTEN mutation. *British Journal of Dermatology* **2006,** *154* (6), 1194-1198.

31. Hobert, J. A.; Embacher, R.; Mester, J. L.; Frazier, T. W., 2nd; Eng, C., Biochemical screening and PTEN mutation analysis in individuals with autism spectrum disorders and macrocephaly. *Eur J Hum Genet* **2014,** *22* (2), 273-6.

32. Schaaf, Christian P.; Zoghbi, Huda Y., Solving the Autism Puzzle a Few Pieces at a Time. *Neuron* **2011,** *70* (5), 806-808.

33. Aspromonte, M. C.; Bellini, M.; Gasparini, A.; Carraro, M.; Bettella, E.; Polli, R.; Cesca, F.; Bigoni, S.; Boni, S.; Carlet, O.; Negrin, S.; Mammi, I.; Milani, D.; Peron, A.; Sartori, S.; Toldo, I.; Soli, F.; Turolla, L.; Stanzial, F.; Benedicenti, F.; Marino-Buslje, C.; Tosatto, S. C. E.; Murgia, A.; Leonardi, E., Characterization of intellectual disability and autism comorbidity through gene panel sequencing. *Hum Mutat* **2019,** *40* (9), 1346-1363.

34. Figer, A.; Kaplan, A.; Frydman, M.; Lev, D.; Paswell, J.; Papa, M. Z.; Goldman, B.; Friedman, E., Germline mutations in the PTEN gene in Israeli patients with Bannayan–Riley–Ruvalcaba syndrome and women with familial breast cancer. *Clinical Genetics* **2002,** *62* (4), 298-302.

35. Iossifov, I.; O'Roak, B. J.; Sanders, S. J.; Ronemus, M.; Krumm, N.; Levy, D.; Stessman, H. A.; Witherspoon, K. T.; Vives, L.; Patterson, K. E.; Smith, J. D.; Paeper, B.; Nickerson, D. A.; Dea, J.; Dong, S.; Gonzalez, L. E.; Mandell, J. D.; Mane, S. M.; Murtha, M. T.; Sullivan, C. A.; Walker, M. F.; Waqar, Z.; Wei, L.; Willsey, A. J.; Yamrom, B.; Lee, Y. H.; Grabowska, E.; Dalkic, E.; Wang, Z.; Marks, S.; Andrews, P.; Leotta, A.; Kendall, J.; Hakker, I.; Rosenbaum, J.; Ma, B.; Rodgers, L.; Troge, J.; Narzisi, G.; Yoon, S.; Schatz, M. C.; Ye, K.; McCombie, W. R.; Shendure, J.; Eichler, E. E.; State, M. W.; Wigler, M., The contribution of de novo coding mutations to autism spectrum disorder. *Nature* **2014,** *515* (7526), 216-21.

36. Ji, X.; Kember, R. L.; Brown, C. D.; Bućan, M., Increased burden of deleterious variants in essential genes in autism spectrum disorder. *Proceedings of the National Academy of Sciences* **2016,** *113* (52), 15054-15059.

37. Krupp, D. R.; Barnard, R. A.; Duffourd, Y.; Evans, S. A.; Mulqueen, R. M.; Bernier, R.; Rivière, J. B.; Fombonne, E.; O'Roak, B. J., Exonic Mosaic Mutations Contribute Risk for Autism Spectrum Disorder. *Am J Hum Genet* **2017,** *101* (3), 369-390.

38. O'Roak, B. J.; Stessman, H. A.; Boyle, E. A.; Witherspoon, K. T.; Martin, B.; Lee, C.; Vives, L.; Baker, C.; Hiatt, J. B.; Nickerson, D. A.; Bernier, R.; Shendure, J.; Eichler, E. E., Recurrent de novo mutations implicate novel genes underlying simplex autism risk. *Nat Commun* **2014,** *5*, 5595.

39. Lim, E. T.; Uddin, M.; De Rubeis, S.; Chan, Y.; Kamumbu, A. S.; Zhang, X.; D'Gama, A. M.; Kim, S. N.; Hill, R. S.; Goldberg, A. P.; Poultney, C.; Minshew, N. J.; Kushima, I.; Aleksic, B.; Ozaki, N.; Parellada, M.; Arango, C.; Penzol, M. J.; Carracedo, A.; Kolevzon, A.; Hultman, C. M.; Weiss, L. A.; Fromer, M.; Chiocchetti, A. G.; Freitag, C. M.; Church, G. M.; Scherer, S. W.; Buxbaum, J. D.; Walsh, C. A., Rates, distribution and implications of postzygotic mosaic mutations in autism spectrum disorder. *Nat Neurosci* **2017,** *20* (9), 1217-1224.

40. Butler, M. G.; Dasouki, M. J.; Zhou, X. P.; Talebizadeh, Z.; Brown, M.; Takahashi, T. N.; Miles, J. H.; Wang, C. H.; Stratton, R.; Pilarski, R.; Eng, C., Subset of individuals with autism spectrum disorders and extreme macrocephaly associated with germline PTEN tumour suppressor gene mutations. *J Med Genet* **2005,** *42* (4), 318-21.

41. Kohno, T.; Takahashi, M.; Fukutomi, T.; Ushio, K.; Yokota, J., Germline mutations of the PTEN/MMAC1 gene in Japanese patients with Cowden disease. *Jpn J Cancer Res* **1998,** *89* (5), 471-4.

42. Bussaglia, E.; Pujol, R. M.; Gil, M. J.; Martí, R. M.; Tuneu, A.; Febrer, M. I.; Garcia-Patos, V.; Ruiz, E. M.; Barnadas, M.; Alegre, M.; Serrano, S.; Matias-Guiu, X., PTEN mutations in eight Spanish families and one Brazilian family with Cowden syndrome. *J Invest Dermatol* **2002,** *118* (4), 639-44.

43. Ruzzo, E. K.; Pérez-Cano, L.; Jung, J. Y.; Wang, L. K.; Kashef-Haghighi, D.; Hartl, C.; Singh, C.; Xu, J.; Hoekstra, J. N.; Leventhal, O.; Leppä, V. M.; Gandal, M. J.; Paskov, K.; Stockham, N.; Polioudakis, D.; Lowe, J. K.; Prober, D. A.; Geschwind, D. H.; Wall, D. P., Inherited and De Novo Genetic Risk for Autism Impacts Shared Networks. *Cell* **2019,** *178* (4), 850-866.e26.

44. Satterstrom, F. K.; Kosmicki, J. A.; Wang, J.; Breen, M. S.; De Rubeis, S.; An, J. Y.; Peng, M.; Collins, R.; Grove, J.; Klei, L.; Stevens, C.; Reichert, J.; Mulhern, M. S.; Artomov, M.; Gerges, S.; Sheppard, B.; Xu, X.; Bhaduri, A.; Norman, U.; Brand, H.; Schwartz, G.; Nguyen, R.; Guerrero, E. E.; Dias, C.; Betancur, C.; Cook, E. H.; Gallagher, L.; Gill, M.; Sutcliffe, J. S.; Thurm, A.; Zwick, M. E.; Børglum, A. D.; State, M. W.; Cicek, A. E.; Talkowski, M. E.; Cutler, D. J.; Devlin, B.; Sanders, S. J.; Roeder, K.; Daly, M. J.; Buxbaum, J. D., Large-Scale Exome Sequencing Study Implicates Both Developmental and Functional Changes in the Neurobiology of Autism. *Cell* **2020,** *180* (3), 568-584.e23.

45. Zhou, X.; Hampel, H.; Thiele, H.; Gorlin, R. J.; Hennekam, R. C.; Parisi, M.; Winter, R. M.; Eng, C., Association of germline mutation in the PTEN tumour suppressor gene and Proteus and Proteus-like syndromes. *Lancet* **2001,** *358* (9277), 210-1.

46. Whitworth, J.; Hoffman, J.; Chapman, C.; Ong, K. R.; Lalloo, F.; Evans, D. G.; Maher, E. R., A clinical and genetic analysis of multiple primary cancer referrals to genetics services. *Eur J Hum Genet* **2015,** *23* (5), 581-7.

47. Shirts, B. H.; Casadei, S.; Jacobson, A. L.; Lee, M. K.; Gulsuner, S.; Bennett, R. L.; Miller, M.; Hall, S. A.; Hampel, H.; Hisama, F. M.; Naylor, L. V.; Goetsch, C.; Leppig, K.; Tait, J. F.; Scroggins, S. M.; Turner, E. H.; Livingston, R.; Salipante, S. J.; King, M. C.; Walsh, T.; Pritchard, C. C., Improving performance of multigene panels for genomic analysis of cancer predisposition. *Genet Med* **2016,** *18* (10), 974-81.

48. Orrico, A.; Galli, L.; Buoni, S.; Orsi, A.; Vonella, G.; Sorrentino, V., Novel PTEN mutations in neurodevelopmental disorders and macrocephaly. *Clin Genet* **2009,** *75* (2), 195-8.

49. De Vivo, I.; Gertig, D. M.; Nagase, S.; Hankinson, S. E.; O'Brien, R.; Speizer, F. E.; Parsons, R.; Hunter, D. J., Novel germline mutations in the PTEN tumour suppressor gene found in women with multiple cancers. *J Med Genet* **2000,** *37* (5), 336-41.

50. McBride, K. L.; Varga, E. A.; Pastore, M. T.; Prior, T. W.; Manickam, K.; Atkin, J. F.; Herman, G. E., Confirmation study of PTEN mutations among individuals with autism or developmental delays/mental retardation and macrocephaly. *Autism Research* **2010,** *3* (3), 137-141.

51. Kersseboom, R.; Dubbink, H. J.; Corver, W. E.; van Tilburg, A. J.; Poley, J. W.; van Leerdam, M. E.; Atmodimedjo, P. N.; van de Laar, I. M.; Collée, J. M.; Dinjens, W. N.; Morreau, H.; Wagner, A., PTEN in colorectal cancer: a report on two Cowden syndrome patients. *Clin Genet* **2012,** *81* (6), 555-62.

52. Sawada, T.; Okada, T.; Miwa, K.; Satoh, H.; Asano, A.; Mabuchi, H., Two novel mutations of PTEN gene in Japanese patients with Cowden syndrome. *American Journal of Medical Genetics Part A* **2004,** *128A* (1), 12-14.

53. Lachlan, K.; Lucassen, A.; Bunyan, D.; Temple, I. K., Cowden syndrome and Bannayan-Riley-Ruvalcaba syndrome represent one condition with variable expression and age-related penetrance: Results of a clinical study of PTEN mutation carriers. *Journal of medical genetics* **2007,** *44*, 579-85.

54. Nizialek, E. A.; Mester, J. L.; Dhiman, V. K.; Smiraglia, D. J.; Eng, C., KLLN epigenotype-phenotype associations in Cowden syndrome. *Eur J Hum Genet* **2015,** *23* (11), 1538-43.

55. Chang, M. T.; Asthana, S.; Gao, S. P.; Lee, B. H.; Chapman, J. S.; Kandoth, C.; Gao, J.; Socci, N. D.; Solit, D. B.; Olshen, A. B.; Schultz, N.; Taylor, B. S., Identifying recurrent mutations in cancer reveals widespread lineage diversity and mutational specificity. *Nat Biotechnol* **2016,** *34* (2), 155-63.

56. Bubien, V.; Bonnet, F.; Brouste, V.; Hoppe, S.; Barouk-Simonet, E.; David, A.; Edery, P.; Bottani, A.; Layet, V.; Caron, O.; Gilbert-Dussardier, B.; Delnatte, C.; Dugast, C.; Fricker, J. P.; Bonneau, D.; Sevenet, N.; Longy, M.; Caux, F.; French Cowden Disease, N., High cumulative risks of cancer in patients with PTEN hamartoma tumour syndrome. *J Med Genet* **2013,** *50* (4), 255-63.

57. O'Roak, B. J.; Vives, L.; Fu, W.; Egertson, J. D.; Stanaway, I. B.; Phelps, I. G.; Carvill, G.; Kumar, A.; Lee, C.; Ankenman, K.; Munson, J.; Hiatt, J. B.; Turner, E. H.; Levy, R.; O'Day, D. R.; Krumm, N.; Coe, B. P.; Martin, B. K.; Borenstein, E.; Nickerson, D. A.; Mefford, H. C.; Doherty, D.; Akey, J. M.; Bernier, R.; Eichler, E. E.; Shendure, J., Multiplex targeted sequencing identifies recurrently mutated genes in autism spectrum disorders. *Science* **2012,** *338* (6114), 1619-22.

58. Tan, W. H.; Baris, H. N.; Burrows, P. E.; Robson, C. D.; Alomari, A. I.; Mulliken, J. B.; Fishman, S. J.; Irons, M. B., The spectrum of vascular anomalies in patients with PTEN mutations: implications for diagnosis and management. *J Med Genet* **2007,** *44* (9), 594-602.

59. Busa, T.; Chabrol, B.; Perret, O.; Longy, M.; Philip, N., Novel PTEN germline mutation in a family with mild phenotype: difficulties in genetic counseling. *Gene* **2013,** *512* (2), 194-7.

60. De Rubeis, S.; He, X.; Goldberg, A. P.; Poultney, C. S.; Samocha, K.; Ercument Cicek, A.; Kou, Y.; Liu, L.; Fromer, M.; Walker, S.; Singh, T.; Klei, L.; Kosmicki, J.; Fu, S.-C.; Aleksic, B.; Biscaldi, M.; Bolton, P. F.; Brownfeld, J. M.; Cai, J.; Campbell, N. G.; Carracedo, A.; Chahrour, M. H.; Chiocchetti, A. G.; Coon, H.; Crawford, E. L.; Crooks, L.; Curran, S. R.; Dawson, G.; Duketis, E.; Fernandez, B. A.; Gallagher, L.; Geller, E.; Guter, S. J.; Sean Hill, R.; Ionita-Laza, I.; Jimenez Gonzalez, P.; Kilpinen, H.; Klauck, S. M.; Kolevzon, A.; Lee, I.; Lei, J.; Lehtimäki, T.; Lin, C.-F.; Ma’ayan, A.; Marshall, C. R.; McInnes, A. L.; Neale, B.; Owen, M. J.; Ozaki, N.; Parellada, M.; Parr, J. R.; Purcell, S.; Puura, K.; Rajagopalan, D.; Rehnström, K.; Reichenberg, A.; Sabo, A.; Sachse, M.; Sanders, S. J.; Schafer, C.; Schulte-Rüther, M.; Skuse, D.; Stevens, C.; Szatmari, P.; Tammimies, K.; Valladares, O.; Voran, A.; Wang, L.-S.; Weiss, L. A.; Jeremy Willsey, A.; Yu, T. W.; Yuen, R. K. C.; Cook, E. H.; Freitag, C. M.; Gill, M.; Hultman, C. M.; Lehner, T.; Palotie, A.; Schellenberg, G. D.; Sklar, P.; State, M. W.; Sutcliffe, J. S.; Walsh, C. A.; Scherer, S. W.; Zwick, M. E.; Barrett, J. C.; Cutler, D. J.; Roeder, K.; Devlin, B.; Daly, M. J.; Buxbaum, J. D.; The, D. D. D. S.; Homozygosity Mapping Collaborative for, A.; Consortium, U. K.; The Autism Sequencing, C., Synaptic, transcriptional and chromatin genes disrupted in autism. *Nature* **2014,** *515* (7526), 209-215.

61. Busch, R. M.; Chapin, J. S.; Mester, J.; Ferguson, L.; Haut, J. S.; Frazier, T. W.; Eng, C., Cognitive characteristics of PTEN hamartoma tumor syndromes. *Genet Med* **2013,** *15* (7), 548-53.

62. Boccone, L.; Dessì, V.; Zappu, A.; Piga, S.; Piludu, M. B.; Rais, M.; Massidda, C.; De Virgiliis, S.; Cao, A.; Loudianos, G., Bannayan-Riley-Ruvalcaba syndrome with reactive nodular lymphoid hyperplasia and autism and a PTEN mutation. *Am J Med Genet A* **2006,** *140* (18), 1965-9.

63. Caux, F.; Plauchu, H.; Chibon, F.; Faivre, L.; Fain, O.; Vabres, P.; Bonnet, F.; Selma, Z. B.; Laroche, L.; Gérard, M.; Longy, M., Segmental overgrowth, lipomatosis, arteriovenous malformation and epidermal nevus (SOLAMEN) syndrome is related to mosaic PTEN nullizygosity. *Eur J Hum Genet* **2007,** *15* (7), 767-73.

64. Yeung, K. S.; Tso, W. W. Y.; Ip, J. J. K.; Mak, C. C. Y.; Leung, G. K. C.; Tsang, M. H. Y.; Ying, D.; Pei, S. L. C.; Lee, S. L.; Yang, W.; Chung, B. H.-Y., Identification of mutations in the PI3K-AKT-mTOR signalling pathway in patients with macrocephaly and developmental delay and/or autism. *Molecular Autism* **2017,** *8* (1), 66.

65. Nelen, M. R.; Kremer, H.; Konings, I. B.; Schoute, F.; van Essen, A. J.; Koch, R.; Woods, C. G.; Fryns, J. P.; Hamel, B.; Hoefsloot, L. H.; Peeters, E. A.; Padberg, G. W., Novel PTEN mutations in patients with Cowden disease: absence of clear genotype-phenotype correlations. *Eur J Hum Genet* **1999,** *7* (3), 267-73.

66. Tammimies, K.; Marshall, C. R.; Walker, S.; Kaur, G.; Thiruvahindrapuram, B.; Lionel, A. C.; Yuen, R. K.; Uddin, M.; Roberts, W.; Weksberg, R.; Woodbury-Smith, M.; Zwaigenbaum, L.; Anagnostou, E.; Wang, Z.; Wei, J.; Howe, J. L.; Gazzellone, M. J.; Lau, L.; Sung, W. W.; Whitten, K.; Vardy, C.; Crosbie, V.; Tsang, B.; D'Abate, L.; Tong, W. W.; Luscombe, S.; Doyle, T.; Carter, M. T.; Szatmari, P.; Stuckless, S.; Merico, D.; Stavropoulos, D. J.; Scherer, S. W.; Fernandez, B. A., Molecular Diagnostic Yield of Chromosomal Microarray Analysis and Whole-Exome Sequencing in Children With Autism Spectrum Disorder. *Jama* **2015,** *314* (9), 895-903.

67. Kirches, E.; Steiner, J.; Schneider, T.; Vorwerk, C. K.; Scherlach, C.; Holtkamp, N.; Keilhoff, G.; Eng, C.; Mawrin, C., Lhermitte–Duclos disease caused by a novel germline PTEN mutation R173P in a patient presenting with psychosis. *Neuropathology and Applied Neurobiology* **2010,** *36* (1), 86-89.

68. Wang, Y.; Tan, X. H.; DiGiovanna, J. J.; Lee, C. C.; Stern, J. B.; Raffeld, M.; Jaffe, E. S.; Kraemer, K. H., Genetic diversity in melanoma metastases from a patient with xeroderma pigmentosum. *J Invest Dermatol* **2010,** *130* (4), 1188-91.

69. LaDuca, H.; Stuenkel, A. J.; Dolinsky, J. S.; Keiles, S.; Tandy, S.; Pesaran, T.; Chen, E.; Gau, C. L.; Palmaer, E.; Shoaepour, K.; Shah, D.; Speare, V.; Gandomi, S.; Chao, E., Utilization of multigene panels in hereditary cancer predisposition testing: analysis of more than 2,000 patients. *Genet Med* **2014,** *16* (11), 830-7.

70. Kim, D. K.; Myung, S. J.; Yang, S. K.; Hong, S. S.; Kim, K. J.; Byeon, J. S.; Lee, G. H.; Kim, J. H.; Min, Y. I.; Lee, S. M.; Jeong, J. Y.; Song, K.; Jung, S. A., Analysis of PTEN gene mutations in Korean patients with Cowden syndrome and polyposis syndrome. *Dis Colon Rectum* **2005,** *48* (9), 1714-22.

71. Staal, F. J.; van der Luijt, R. B.; Baert, M. R.; van Drunen, J.; van Bakel, H.; Peters, E.; de Valk, I.; van Amstel, H. K.; Taphoorn, M. J.; Jansen, G. H.; van Veelen, C. W.; Burgering, B.; Staal, G. E., A novel germline mutation of PTEN associated with brain tumours of multiple lineages. *Br J Cancer* **2002,** *86* (10), 1586-91.

72. C Yuen, R. K.; Merico, D.; Bookman, M.; L Howe, J.; Thiruvahindrapuram, B.; Patel, R. V.; Whitney, J.; Deflaux, N.; Bingham, J.; Wang, Z.; Pellecchia, G.; Buchanan, J. A.; Walker, S.; Marshall, C. R.; Uddin, M.; Zarrei, M.; Deneault, E.; D'Abate, L.; Chan, A. J. S.; Koyanagi, S.; Paton, T.; Pereira, S. L.; Hoang, N.; Engchuan, W.; Higginbotham, E. J.; Ho, K.; Lamoureux, S.; Li, W.; MacDonald, J. R.; Nalpathamkalam, T.; Sung, W. W. L.; Tsoi, F. J.; Wei, J.; Xu, L.; Tasse, A.-M.; Kirby, E.; Van Etten, W.; Twigger, S.; Roberts, W.; Drmic, I.; Jilderda, S.; Modi, B. M.; Kellam, B.; Szego, M.; Cytrynbaum, C.; Weksberg, R.; Zwaigenbaum, L.; Woodbury-Smith, M.; Brian, J.; Senman, L.; Iaboni, A.; Doyle-Thomas, K.; Thompson, A.; Chrysler, C.; Leef, J.; Savion-Lemieux, T.; Smith, I. M.; Liu, X.; Nicolson, R.; Seifer, V.; Fedele, A.; Cook, E. H.; Dager, S.; Estes, A.; Gallagher, L.; Malow, B. A.; Parr, J. R.; Spence, S. J.; Vorstman, J.; Frey, B. J.; Robinson, J. T.; Strug, L. J.; Fernandez, B. A.; Elsabbagh, M.; Carter, M. T.; Hallmayer, J.; Knoppers, B. M.; Anagnostou, E.; Szatmari, P.; Ring, R. H.; Glazer, D.; Pletcher, M. T.; Scherer, S. W., Whole genome sequencing resource identifies 18 new candidate genes for autism spectrum disorder. *Nature Neuroscience* **2017,** *20* (4), 602-611.

73. Du, X.; Gao, X.; Liu, X.; Shen, L.; Wang, K.; Fan, Y.; Sun, Y.; Luo, X.; Liu, H.; Wang, L.; Wang, Y.; Gong, Z.; Wang, J.; Yu, Y.; Li, F., Genetic Diagnostic Evaluation of Trio-Based Whole Exome Sequencing Among Children With Diagnosed or Suspected Autism Spectrum Disorder. *Front Genet* **2018,** *9*, 594.

74. Allen, A. S.; Berkovic, S. F.; Cossette, P.; Delanty, N.; Dlugos, D.; Eichler, E. E.; Epstein, M. P.; Glauser, T.; Goldstein, D. B.; Han, Y.; Heinzen, E. L.; Hitomi, Y.; Howell, K. B.; Johnson, M. R.; Kuzniecky, R.; Lowenstein, D. H.; Lu, Y. F.; Madou, M. R.; Marson, A. G.; Mefford, H. C.; Esmaeeli Nieh, S.; O'Brien, T. J.; Ottman, R.; Petrovski, S.; Poduri, A.; Ruzzo, E. K.; Scheffer, I. E.; Sherr, E. H.; Yuskaitis, C. J.; Abou-Khalil, B.; Alldredge, B. K.; Bautista, J. F.; Berkovic, S. F.; Boro, A.; Cascino, G. D.; Consalvo, D.; Crumrine, P.; Devinsky, O.; Dlugos, D.; Epstein, M. P.; Fiol, M.; Fountain, N. B.; French, J.; Friedman, D.; Geller, E. B.; Glauser, T.; Glynn, S.; Haut, S. R.; Hayward, J.; Helmers, S. L.; Joshi, S.; Kanner, A.; Kirsch, H. E.; Knowlton, R. C.; Kossoff, E. H.; Kuperman, R.; Kuzniecky, R.; Lowenstein, D. H.; McGuire, S. M.; Motika, P. V.; Novotny, E. J.; Ottman, R.; Paolicchi, J. M.; Parent, J. M.; Park, K.; Poduri, A.; Scheffer, I. E.; Shellhaas, R. A.; Sherr, E. H.; Shih, J. J.; Singh, R.; Sirven, J.; Smith, M. C.; Sullivan, J.; Lin Thio, L.; Venkat, A.; Vining, E. P.; Von Allmen, G. K.; Weisenberg, J. L.; Widdess-Walsh, P.; Winawer, M. R., De novo mutations in epileptic encephalopathies. *Nature* **2013,** *501* (7466), 217-21.

75. Negishi, Y.; Miya, F.; Hattori, A.; Johmura, Y.; Nakagawa, M.; Ando, N.; Hori, I.; Togawa, T.; Aoyama, K.; Ohashi, K.; Fukumura, S.; Mizuno, S.; Umemura, A.; Kishimoto, Y.; Okamoto, N.; Kato, M.; Tsunoda, T.; Yamasaki, M.; Kanemura, Y.; Kosaki, K.; Nakanishi, M.; Saitoh, S., A combination of genetic and biochemical analyses for the diagnosis of PI3K-AKT-mTOR pathway-associated megalencephaly. *BMC Med Genet* **2017,** *18* (1), 4.

76. Wu, H.; Li, H.; Bai, T.; Han, L.; Ou, J.; Xun, G.; Zhang, Y.; Wang, Y.; Duan, G.; Zhao, N.; Chen, B.; Du, X.; Yao, M.; Zou, X.; Zhao, J.; Hu, Z.; Eichler, E. E.; Guo, H.; Xia, K., Phenotype-to-genotype approach reveals head-circumference-associated genes in an autism spectrum disorder cohort. *Clin Genet* **2020,** *97* (2), 338-346.

77. Banneau, G.; Guedj, M.; MacGrogan, G.; de Mascarel, I.; Velasco, V.; Schiappa, R.; Bonadona, V.; David, A.; Dugast, C.; Gilbert-Dussardier, B.; Ingster, O.; Vabres, P.; Caux, F.; de Reynies, A.; Iggo, R.; Sevenet, N.; Bonnet, F.; Longy, M., Molecular apocrine differentiation is a common feature of breast cancer in patients with germline PTEN mutations. *Breast Cancer Res* **2010,** *12* (4), R63.

78. Chi, S.-G.; Kim, H.-J.; Park, B.-J.; Min, H.-J.; Park, J.-H.; Kim, Y.-W.; Dong, S.-H.; Kim, B.-H.; Lee, J.-I.; Chang, Y.-W.; Chang, R.; Kim, W.-K.; Yang, M.-H., Mutational abrogation of the <em>PTEN/MMAC1</em> gene in gastrointestinal polyps in patients with Cowden disease. *Gastroenterology* **1998,** *115* (5), 1084-1089.

79. Ueno, Y.; Enokizono, T.; Fukushima, H.; Ohto, T.; Imagawa, K.; Tanaka, M.; Sakai, A.; Suzuki, H.; Uehara, T.; Takenouchi, T.; Kosaki, K.; Takada, H., A novel missense PTEN mutation identified in a patient with macrocephaly and developmental delay. *Hum Genome Var* **2019,** *6*, 25.

80. Sawada, T.; Hamano, N.; Satoh, H.; Okada, T.; Takeda, Y.; Mabuchi, H., Mutation analysis of the PTEN / MMAC1 gene in Japanese patients with Cowden disease. *Jpn J Cancer Res* **2000,** *91* (7), 700-5.
